# Grouping of complex substances using analytical chemistry data: A framework for quantitative evaluation and visualization

**DOI:** 10.1371/journal.pone.0223517

**Published:** 2019-10-10

**Authors:** Melis Onel, Burcu Beykal, Kyle Ferguson, Weihsueh A. Chiu, Thomas J. McDonald, Lan Zhou, John S. House, Fred A. Wright, David A. Sheen, Ivan Rusyn, Efstratios N. Pistikopoulos

**Affiliations:** 1 Artie McFerrin Department of Chemical Engineering, Texas A&M University, College Station, TX, United States of America; 2 Texas A&M Energy Institute, Texas A&M University, College Station, TX, United States of America; 3 Department of Veterinary Integrative Biosciences, Texas A&M University, College Station, TX, United States of America; 4 Department of Environmental and Occupational Health, Texas A&M University, College Station, TX, United States of America; 5 Department of Statistics, Texas A&M University, College Station, TX, United States of America; 6 Bioinformatics Research Center, North Carolina State University, Raleigh, NC, United States of America; 7 Departments of Statistics and Biological Sciences, North Carolina State University, Raleigh, NC, United States of America; 8 Chemical Sciences Division, National Institute of Standards and Technology, Gaithersburg, MD, United States of America; Newcastle University, UNITED KINGDOM

## Abstract

A detailed characterization of the chemical composition of complex substances, such as products of petroleum refining and environmental mixtures, is greatly needed in exposure assessment and manufacturing. The inherent complexity and variability in the composition of complex substances obfuscate the choices for their detailed analytical characterization. Yet, in lieu of exact chemical composition of complex substances, evaluation of the degree of similarity is a sensible path toward decision-making in environmental health regulations. Grouping of similar complex substances is a challenge that can be addressed via advanced analytical methods and streamlined data analysis and visualization techniques. Here, we propose a framework with unsupervised and supervised analyses to optimally group complex substances based on their analytical features. We test two data sets of complex oil-derived substances. The first data set is from gas chromatography-mass spectrometry (GC-MS) analysis of 20 Standard Reference Materials representing crude oils and oil refining products. The second data set consists of 15 samples of various gas oils analyzed using three analytical techniques: GC-MS, GC×GC-flame ionization detection (FID), and ion mobility spectrometry-mass spectrometry (IM-MS). We use hierarchical clustering using Pearson correlation as a similarity metric for the unsupervised analysis and build classification models using the Random Forest algorithm for the supervised analysis. We present a quantitative comparative assessment of clustering results via Fowlkes–Mallows index, and classification results via model accuracies in predicting the group of an unknown complex substance. We demonstrate the effect of (i) different grouping methodologies, (ii) data set size, and (iii) dimensionality reduction on the grouping quality, and (iv) different analytical techniques on the characterization of the complex substances. While the complexity and variability in chemical composition are an inherent feature of complex substances, we demonstrate how the choices of the data analysis and visualization methods can impact the communication of their characteristics to delineate sufficient similarity.

## 1. Introduction

Products of petroleum refining are prototypical UVCB (Unknown or Variable composition, Complex reaction products and Biological materials) substances [1]. UVCBs are some of the most challenging substances for the industry and regulators, because there are few established frameworks for evaluating UVCBs under current chemical regulatory policy and ensuring that there is no underestimation of hazard to either workers or the general users of the end-products [2]. Indeed, the complexity of the chemical composition of petroleum substances [3, 4], and in particular their multi-constituent nature and variability in product composition based on the variability in crude oil stocks, poses unique challenges to the regulators and registrants of these substances [5].

Typically, individual UVCB substances are assigned into a product category based on the manufacturing processes, physical/chemical properties (including refining history and boiling point/carbon number ranges), and limited analytical chemical information (such as hydrocarbon classes) [1, 2]. However, such broad similarity parameters may not always be considered sufficient by the regulatory bodies, and new approaches to facilitate the grouping of UVCBs are needed [6]. Recent developments in high-resolution and multi-dimensional analytical techniques improve characterization of complex substances by providing greater resolution of their chemical composition [7, 8]. Despite these advances, full chemical characterization of complex substances, such as petroleum UVCB substances, is still largely unattainable [6]. This presents a challenge for defining “sufficient similarity” for a substance of interest in comparison to those substances that may have already been tested for their potential human and ecological effects [9, 10].

**Fig 1:**
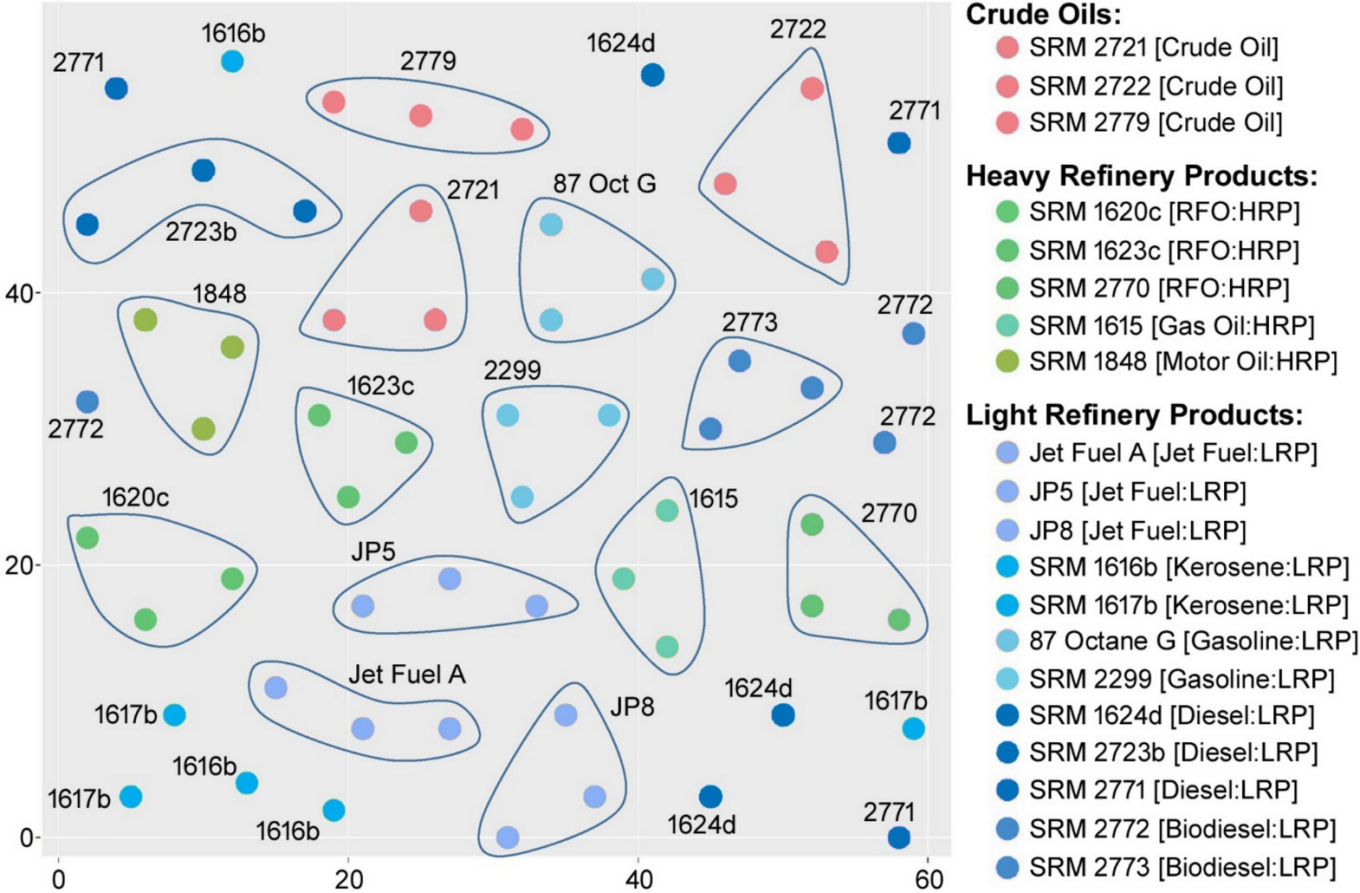
SOM recreated from de Carvalho Rocha, Schantz (14).

A variety of analytical methods can be used to rapidly profile chemical composition of environmental samples and UVCBs, and all of them produce complex high-dimensional data sets [6, 11]. Quantitative interpretation of high-dimensional data has been an active area of statistics and a number of algorithms have been applied to classify unknown samples, or to derive discriminating data features [8, 12]. For example, data integration, clustering and visualization techniques using ion mobility-mass spectrometry (IM-MS) data of a subset of UVCBs have been used to determine the group-specific similarities [13]. Comparative analyses have also been performed. For example, de Carvalho Rocha, Schantz [14] has utilized principal components analysis (MPCA), principal factors analysis (PARAFAC), and self-organizing maps (SOM) analysis to differentiate among various types of fuels via pattern recognition. Although SOMs produce visually appealing grouping maps (Fig 1), comparative assessment to determine the optimal grouping is a challenge [15, 16]. Additional pattern recognition analysis techniques [1, 17–19] have also been explored to interpret the patterns in complex data sets; however, the outcomes of these methods are largely qualitative in nature and rely on the subjective visual evaluation of the grouping outcomes rather than quantitative comparative metrics.

The work presented in this manuscript aims to bridge the gap between the quantitative evaluation and visual communication of the grouping analysis outcomes to find the optimal grouping of complex substances. In this study, we present a data-driven framework that includes two separate workflows for grouping of complex chemical substances. First, we present an unsupervised data analysis workflow based on hierarchical clustering, where the results are demonstrated through dendrograms, and the grouping quality is evaluated against existing manufacturing classes using the Fowlkes-Mallows (F-M) index. Second, we use supervised analysis. We have applied the Random Forest algorithm to train classification models to predict the manufacturing category information of these substances. The overall premise of the proposed framework is to provide (i) optimal grouping of complex substances, (ii) improved interpretation of the grouping results for decision-makers with the use of visualization techniques and identification of the most informative features, and (iii) comparative assessment of the grouping results by reporting quantitative metrics (i.e. the Fowlkes-Mallows index for clustering, and accuracy for classification analysis).

## 2. Materials & methods

### 2.1. Materials

In this study, we used two different sets of benchmark analytical chemistry data of: (i) 3 replicates of 20 Standard Reference Materials (SRM) (Table 1), and (ii) several petroleum UVCB substances, which were supplied by the European Petroleum Refiners Association AISBL, Concawe division (Brussels, Belgium) and referred to as “Petroleum UVCB samples” (Table 2). Specifically, SRMs are petroleum-related Certified Reference Materials and provided by the National Institute of Standards and Technology (NIST) [14]. In contrast, Petroleum UVCB samples were obtained from three separate refinement processes and categorized as straight run gas oils (SRGOs), other gas oils (OGOs), and vacuum and hydro-treated gas oils (VHGOs). Polycyclic-aromatic hydrocarbon (PAH), saturated hydrocarbon, and crude oil standards were provided by the Texas A&M Geochemical and Environmental Research Group (GERG) (College Station, TX).

**Table 1:** Standard Reference Materials (SRM) samples from de Carvalho Rocha, Schantz (14).

SRM ID	3-Class Grouping	9-Class Grouping	16-Class Grouping	Sample IDs
SRM 2722	Crude Oil	Crude Oil	Crude Oil (Heavy-Sweet)	petro203; petro204; petro205
SRM 2721	Crude Oil	Crude Oil	Crude Oil (Light-Sour)	petro274; petro275; petro276
SRM 2779	Crude Oil	Crude Oil	Gulf of Mexico Crude Oil	petro270; petro271; petro272
SRM 1615	Heavy Refinery Product	Gas Oil	Gas Oil	petro207; petro208; petro209
SRM 1848	Heavy Refinery Product	Motor Oil	Motor Oil Additive	petro218; petro219; petro220
SRM 2770	Heavy Refinery Product	RFO	S in Residual Fuel Oil	petro234; petro235; petro236
SRM 1623c	Heavy Refinery Product	RFO	S in Residual Fuel Oil	petro238; petro239; petro240
SRM 1620c	Heavy Refinery Product	RFO	S in Residual Fuel Oil	petro278; petro279; petro280
SRM 2773	Light Refinery Product	Biodiesel	Biodiesel (Animal-based)	petro230; petro231; petro232
SRM 2772	Light Refinery Product	Biodiesel	Biodiesel (Soy-based)	petro266; petro267; petro268
SRM 2723b	Light Refinery Product	Diesel	Low S Diesel	petro226; petro227; petro228
SRM 1624d	Light Refinery Product	Diesel	Sulfur in Diesel	petro214; petro215; petro216
SRM 2771	Light Refinery Product	Diesel	Zero S Diesel	petro222; petro223; petro224
Gasoline	Light Refinery Product	Gasoline	87 Octane Gasoline	petro258; petro259; petro260
SRM 2299	Light Refinery Product	Gasoline	S in gasoline	petro210; petro211; petro212
JP8	Light Refinery Product	Jet Fuel	Jet Fuel	petro246; petro247; petro248
JP5	Light Refinery Product	Jet Fuel	Jet Fuel	petro250; petro251; petro252
Jet Fuel A	Light Refinery Product	Jet Fuel	Jet Fuel	petro254; petro255; petro256
SRM 1617b	Light Refinery Product	Kerosene	S in Kerosene (High Level)	petro242; petro243; petro244
SRM 1616b	Light Refinery Product	Kerosene	S in Kerosene (Low Level)	petro262; petro263; petro264

*16-class grouping is based on designation by the National Institute of Standards and Technology (NIST), which was further grouped into 9 major classes. The 3-class grouping reflects the major refining distinctions among the SRMs.

**Table 2:** Petroleum UVCB samples.

Sample ID	Manufacturing class	CAS RN	CAS Name
CON07	OGO	64742-46-7	Distillates (petroleum), hydrotreated middle
CON09	OGO	64742-80-9	Distillates (petroleum), hydro-desulfurized middle
CON01	SRGO	64741-43-1	Gas oils (petroleum), straight-run
CON05	SRGO		
CON02	SRGO	68814-87-9	Distillates (petroleum), full-range straight-run middle
CON03	SRGO		
CON04	SRGO	68915-96-8	Distillates (petroleum), heavy straight-run
CON12	VHGO	64741-49-7	Condensates (petroleum), vacuum tower
CON13	VHGO	64741-58-8	Gas oils (petroleum), light vacuum
CON14	VHGO	64741-77-1	Distillates (petroleum), light hydrocracked
CON15	VHGO	64742-87-6	Gas oils (petroleum), hydrodesulfurized light vacuum
CON16	VHGO	68334-30-5	Fuels, diesel
CON17	VHGO	68476-30-2	Fuel oil, no. 2
CON18	VHGO	68476-31-3	Fuel oil, no. 4
CON20	VHGO	92045-24-4	Gas oils (petroleum), hydrotreated light vacuum

### 2.2. Chemical fingerprinting and experimental data processing

The analytical chemistry profile of SRMs was derived via Gas Chromatography-Mass Spectrometry (GC-MS) [14], whereas the chemical fingerprint of Petroleum UVCB substances was assessed with 3 different analytical chemistry techniques: (i) comprehensive two-dimensional gas chromatography with flame ionization detector (GC×GC-FID), (ii) GC-MS, and (iii) Ion Mobility Mass Spectrometry (IM-MS). The detailed experimental procedure is provided in Ferguson [20].

The GC-MS data from de Carvalho Rocha, Schantz (14) is a three-dimensional array, which consists of 23,248 elution times, and the 301 masses in the mass spectra for 60 Standard Reference Materials (triplicate runs of 20 samples). To reduce the computational complexity of the grouping analysis and the noise in the GC-MS data, we have selected 55 out of 301 m/z values (i.e. analytes) that correspond to Polycyclic Aromatic Hydrocarbons (PAHs) (S1 Table) and summed over the entire elution time dimension. This yields a two-dimensional (60 × 55) array, which is then used for grouping analysis.

### 2.3. Data analysis and visualization framework

**Fig 2:**
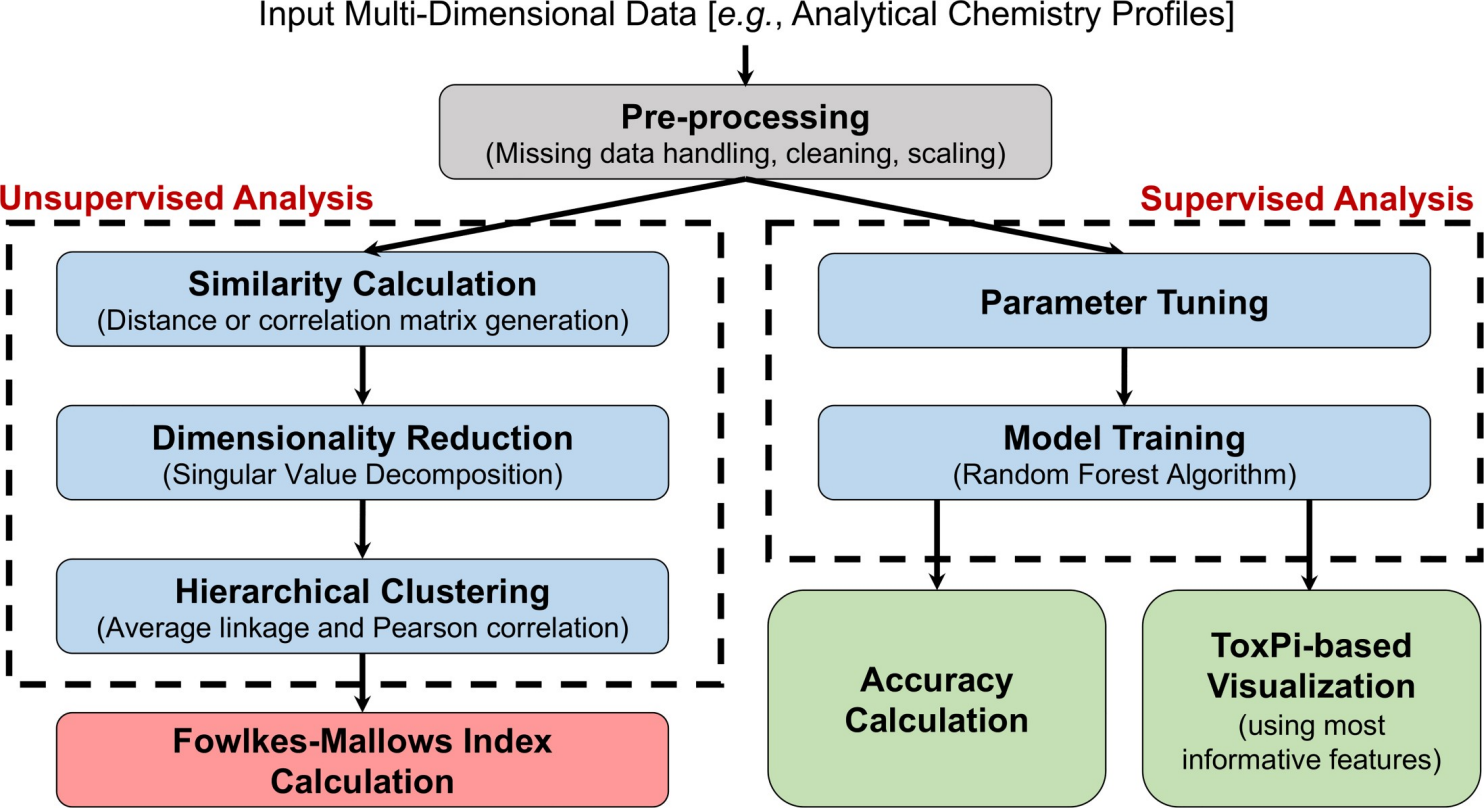
Data processing and visualization workflow.

We used two analysis workflows for grouping complex substances (Fig 2). In the unsupervised analysis, complex substances are grouped based on the similarity between the characteristics (i.e. analytical chemistry profiles) of the samples (complex substances) without prior knowledge of sample labels or categories. To evaluate the outcome of such grouping, we included a quantitative metric into the unsupervised analysis workflow to compare the outcome to a previously reported categorization of the samples (i.e. manufacturing classes). The details of the proposed unsupervised analysis workflow are described in Section 2.3.2. In the supervised analysis, known categorizations/classes of the samples are used to build classification models, which can then be used to predict the class for an unknown substance. This idea is based on the read-across, where similar complex substances that are grouped together according to their physical/chemical properties may have similar effects [2]. The details of the proposed unsupervised analysis workflow are described in Section 2.3.3. Independent of which workflow (unsupervised or supervised analysis), the initial common step is data pre-processing, which is crucial to obtain robust and reliable grouping models (Section 2.3.1).

#### 2.3.1. Data pre-processing

Data pre-processing steps include (i) data formatting, (ii) handling missing data, and (iii) data cleaning and scaling. Following these steps ensures data quality in order to build robust and reliable models. The application of these steps to each specific data set is provided below.

First the three-dimensional analytical chemistry data (the GC×GC-FID, GC-MS, and IM-MS data sets) needed to be unfolded into a 2-dimensional matrix. The GC-MS data of SRMs, after the experimental data processing step described in Section 2.2, is already two-dimensional. The unfolding was performed so that, for the final matrix, rows correspond to the complex substances, and columns are the analytical features (measurements), which are the concatenated values of carbon number and molecular class composition. This process yielded an array size of 15 × 310 for GC×GC-FID, 15 × 248 for GC-MS, and 15 × 403 for IM-MS data sets of the Petroleum UVCB samples.

Next, the two-dimensional analytical chemistry data sets were examined to detect any missing points. Although advanced missing data handling methods are sometimes used in complex data analysis [21], here the missing data points within analytical chemistry profiles indicate undetected chemical composition for a specific molecular class. Thus, we replaced the corresponding missing fields with zeros.

The data sets were further cleaned by removing the columns (carbon number–molecular class compositions) if they show negligible variation. Here, we removed columns with a standard deviation (SD) of 0, or SD<0.05 for the smaller Petroleum UVCB data set. This step reduced the number of features in GC×GC-FID from 310 to 192, GC-MS from 248 to 62, and IM-MS data from 403 to 68. This step did not eliminate any measurements from the 60 x 55 matrix of SRM samples.

The final step prior to data analysis is the scaling of the data sets. The clean two-dimensional arrays were scaled by using row-wise min-max scaling, where each row corresponds to a new sample and each column is a new analytical feature. Each row value was scaled by subtracting the minimum value of that row and then dividing it by the range of the corresponding row. Row-wise scaling was not performed on Petroleum UVCB data sets, because the data was already pre-processed within PetroOrg software [22] and row-wise scaled. Prior to the classification analysis, we also performed column-wise min-max scaling on the row-wise scaled data arrays. This additional scaling step is required to ensure that each measurement has approximately equal weight in training classification models.

#### 2.3.2. Unsupervised analysis workflow

Unsupervised analysis examines the patterns of data to draw conclusions for the grouping structure of the samples without the reference categorization information. The two most prevalent unsupervised analysis techniques used in the literature are clustering analysis [23], as used in our workflow (Fig 2 left panel), and SOMs [24]. The detailed steps of this workflow are given below. The R Markdown documentation of this analysis for SRM samples is also provided in the Supplementary Material (S1 Text).

Under the unsupervised workflow, we performed cluster analyses using the data as described, and also after producing a reduced-rank data set, in order to judge the effect of using reduced-rank data (i.e., after “de-noising” the data). The original unscaled SRM and Petroleum UVCB data sets were linearly scaled and centered in a row-wise fashion (i.e. z-score normalization). The resultant data was then decomposed using singular value decomposition (SVD) [25, 26] in R to produce a reduced-rank data set corresponding to 85% of the variation in the original data.

Pearson correlations of both the original and reduced-rank analytical chemistry data were used as a similarity index for hierarchical cluster analysis of the samples using hclust in R with average linkage [27, 28].

A quantitative comparison of the clustering to the known substance categories (treated here as a “gold standard”) was performed using the Fowlkes-Mallows (F-M) index [29]. The F-M index is traditionally used to compare two dendrograms but can also be used to compare a single dendrogram to a fixed categorization. We created two sets of hierarchical clustering dendrograms for both the Petroleum UVCB and SRM samples. First, an artificial dendrogram was generated by calculating the Euclidean distance between the indices (0/1) of a reference categorization. Second, the correlation matrices of the Petroleum UVCB and SRM samples were used to generate a dendrogram. Next, the dendrograms were both cut into the known number of manufacturing classes (i.e. 3 for Petroleum UVCB samples, and 3, 9, or 16 for SRM samples) to assess the number of the common complex substances in the obtained clusters. This number was then used to calculate the F-M index for the two groupings (i.e., comparing known categorization to the data-based grouping). The F-M index can be expressed as the geometric mean of precision and recall, two machine learning metrics that are widely used in data-driven modeling [23]. Expressed mathematically, we have

$$FM=\sqrt{\frac{TP}{TP+FP}.\frac{TP}{TP+FN}}$$

where TP is True Positive, FP is False Positive, and FN is False Negative. TP indicates the number of complex substances that are grouped under category A in terms of manufacturing category and are also grouped under category A in terms of analytical chemistry profile. In contrast, FP and FN denote the number of complex substances that are grouped differently. The F-M index varies between 0 and 1, where 0 indicates the absence of any similarity, and 1 indicates 100% identity between reference categorization and clustering results. More details on the F-M index and other metrics for clustering comparison can be found in Wagner & Wagner [30]. The F-M index was calculated via the FM_index function of the dendextend package in R.

To test the statistical significance of the grouping results, we also calculated the distribution of the F-M index under the null hypothesis of no relation between two clustering dendrograms. This null distribution was generated by shuffling the group labels of samples using 1000 permutations, with an empirical *p*-value determined by the proportion of permuted F-M index values exceeding the observed. We used α = 0.05 as a false-positive threshold. The null F-M index calculation with 1000 permutations of the group labels was performed via the Bk_permutations function of the dendextend package in R.

#### 2.3.3. Supervised analysis workflow

Although unsupervised analysis can elucidate previously unknown structures in the data, supervised methods can identify the features most influential in classification. Moreover, in this context, supervised analysis may highlight substances that show comparatively poor similarity to the other members of the manufacturing category. Supervised learning algorithms are widely used in various engineering and sciences problems [31–40]. Here, we used the Random Forest decision-tree algorithm [41] to train models to predict manufacturing category from the features. The models were evaluated by their classification accuracy, and the results were visualized via ToxPi representation [42] for enhanced interpretation (Fig 2 right). The steps of the proposed supervised analysis workflow are provided below and applied to both Petroleum UVCB and SRM data sets. The documentation of the analysis through SRM samples was created using R Markdown and provided in the Supplementary Material (S1 Text).

In our implementation of the Random Forests, the number of analytical features was tuned via grid search using the trainControl function of the caret package in R, where each model training was performed using leave-one-out cross validation with 500 decision trees. The final Random Forest classifier was then built on the whole data set with 500 decision trees, where each tree was modeled by using the optimal number of analytical features. In addition, the ranking of the analytical features was obtained by calculating the mean decrease in classification accuracy among the 500 decision trees. This analysis was done via the randomForest function of the randomForest package.

To evaluate the classification model accuracy, an initial step was to extract the confusion matrix of the model, i.e. the number of true and falsely predicted samples for each class. Next, the classification accuracy was calculated, which is the percentage of true predicted number of samples from all classes with respect to the total number of samples.

In addition to the quantification of the classification models, we produced Toxicological Prioritization Index (ToxPi) profiles of complex substances by using the ranked analytical feature list from the classification analysis [42–44]. By integrating multiple data sources into an overall, weight-of-evidence score, and transforming them into clear visual rankings, ToxPi provides an effective way for visual communication of high-dimensional data sets. Here we integrated the top 10 most informative chromatographic features that were extracted during classification modeling step, to obtain the ToxPi visualization of complex substances.

## 3. Results & discussion

### 3.1. Quantifying the Unsupervised analysis

A recent study [14] has shown that GC-MS combined with unsupervised chemometric analysis can be used to differentiate among complex substances and mixtures. The authors have concluded that the SOM non-linear method proved to be effective in generating a separation model. However, the model is more difficult to interpret than the linear models such as MPCA and PARAFAC [45, 46]. The unified distance matrix of the SOM analysis of the 20 SRMs (Table 1) from de Carvalho Rocha, Schantz (14) is shown in Fig 1. The visualization of the data using SOM makes it apparent that the replicates of the same sample were clustered well (15 of 20 samples have all 3 replicates in close proximity to each other) (Fig 1). However, it is less obvious that the SOM analysis can discriminate among the broader categories of samples (3 classes: crude oils, heavy and light refinery products; 9 classes: crude oils, residual fuel oils, gas oil, motor oil, biodiesels, diesels, gasolines, kerosenes and jet fuels). Only jet fuels and gasoline samples of light refinery products were clustered close to each other (Fig 1).

**Fig 3:**
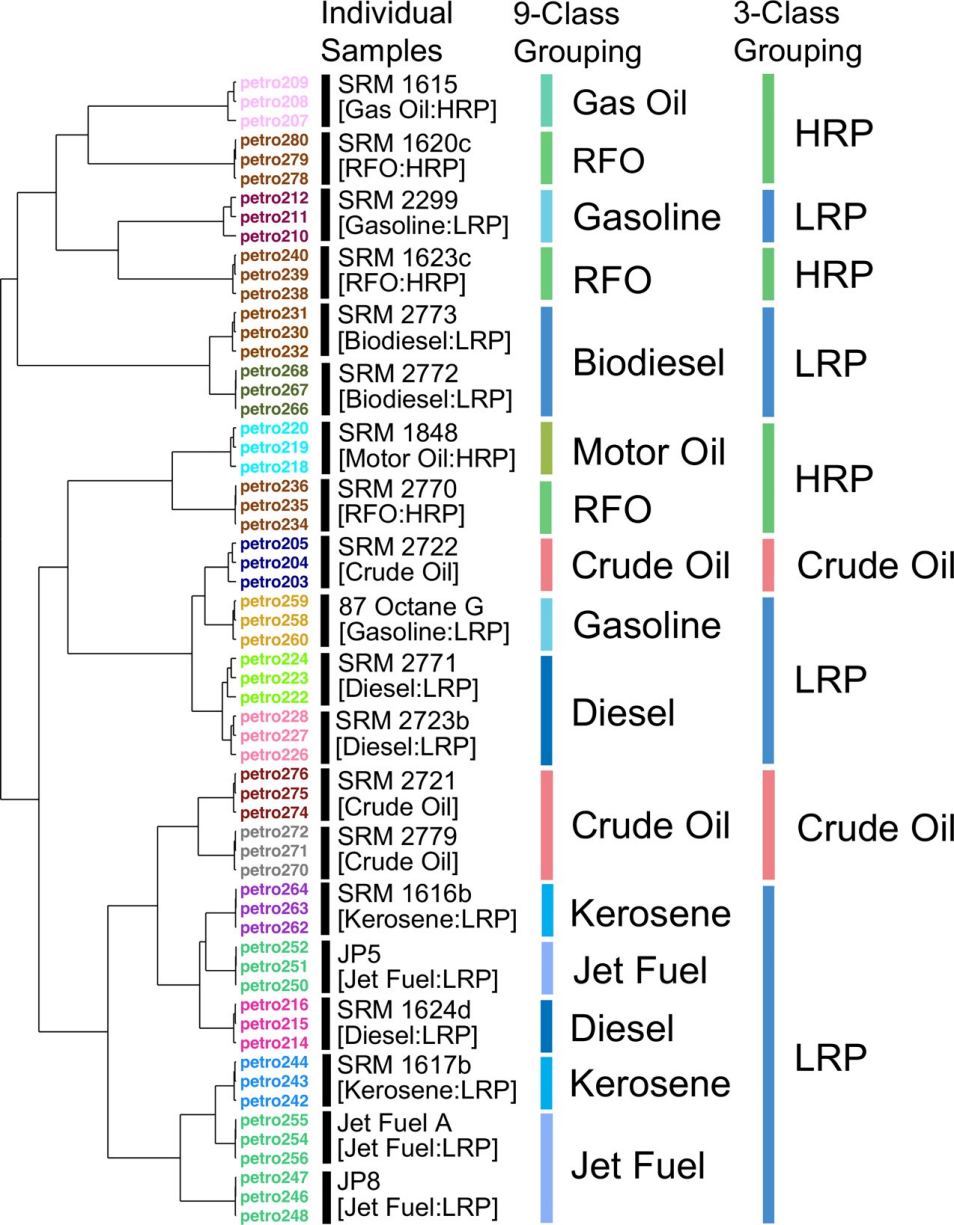
Dendrograms for the SRM samples clustering from the reduced data set into 3, 9 and 16 categories. LRP: Light Refinery Product, HRP: Heavy Refinery Product.

To explore additional visualization methods, we used the data from de Carvalho Rocha, Schantz (14) to perform unsupervised clustering analysis of the samples (Fig 3). The results showed that all technical replicates of 20 substances were clustered tightly, which indicates high reproducibility of the analytical data from GC-MS analysis of these complex substances. However, when 3 or 9 broader manufacturing classes were considered, the samples were not clustered as closely as they were in the 16 manufacturing classes. For 9 class grouping, replicate samples of gas oils, biodiesels, and motor oils were grouped together in distinct clusters. In 3 class grouping results, only crude oil samples were grouped under one of the three clusters. Even though most of the light refinery products (one gasoline, three diesel, two jet fuels, and two kerosene samples) were clustered together in one of the three groups, one gasoline and two biodiesel samples fall into separate clusters (Fig 3). These analyses demonstrated that the analytical features derived from GC-MS were, by themselves, insufficient for justifying grouping of these complex substances into the manufacturing categories.

Next, we aimed to quantitatively compare the outcomes of SOM and clustering analyses to the manufacturing class categories of these samples. We used the Fowlkes-Mallows (F-M) index to provide a quantitative metric for such comparisons [29]. Although there is no direct method to assess the grouping quality using the SOM analysis, we have extracted the x and y coordinates of each SRM sample on the SOM map as reported by de Carvalho Rocha, Schantz (14) (Fig 1) and used the Euclidean distance-based similarity matrix to obtain the F-M index for the SOM-based grouping analysis. The F-M index was also used to assess the effect of dimensionality reduction on the outcomes of clustering analyses.

**Fig 4:**
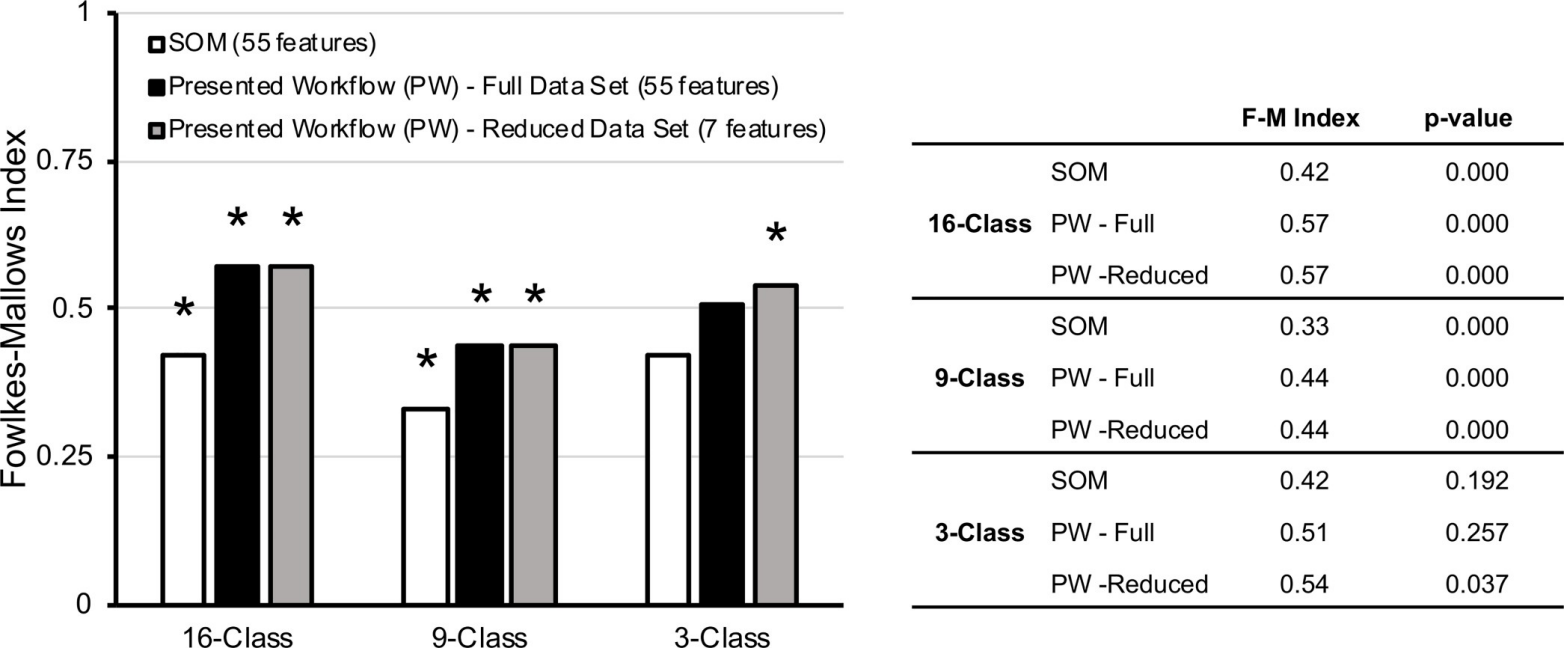
Fowlkes-Mallows index for the outcomes of clustering of SRM samples. * indicates that the results are statistically significant at the 0.05 level.

Fig 4 displays the F-M indices for SOM-based analysis, as well as the presented unsupervised analysis workflow on full data set of 55 GC-MS features and a reduced set of 7 features after SVD. The *p-*values for the significance of the correspondence of the clustering compared to the known class assignment are also reported (Fig 4). The *p-*values obtained for 3-class grouping were higher than 0.05 for the SOM-based and original data set of SRM samples, implying these results were not statistically significant. Subtle differences differentiate these materials into 16 categories. When grouped under 3 categories, these differences presented themselves as noise. Hence, the random permutation of these samples led to higher F-M indices by chance. In contrast, the *p-*values for 3-class grouping with the reduced data sets were lower than 0.05. This indicates evidence that dimensionality reduction eliminates redundant analytical features from the data sets (from 55 to 7) which further reduces the noise, leading to statistically significant results with a similar (and slightly improved) F-M index.

Based on 9 and 16-class groupings of SRMs, one can clearly observe that hierarchical clustering outperformed SOM analysis. The F-M index increased from 0.33 to 0.44, and 0.42 to 0.57 for 9 and 16-class groupings, respectively. Although the dimensionality reduction did not further increase the F-M index for 9 and 16 class groupings, it also did not hinder the grouping quality and provided equally good results with lower number features (7 out of 55).

### 3.2. Importance of substance sample size during supervised analysis

Here, we benefit from the read-across hypothesis that “complex substances that group similarly based on manufacturing may exhibit similar hazard profiles,” and move from unsupervised to supervised analysis. To this end, we are building classification models using analytical chemistry profiles of samples. For each of the 20 SRM substances, GC-MS was run three times, which made the final GC-MS data set larger in terms of the number of samples. Thus, an interesting question that we can examine is that how many sample replicates would be adequate to develop data-driven models that can precisely differentiate class patterns.

**Fig 5:**
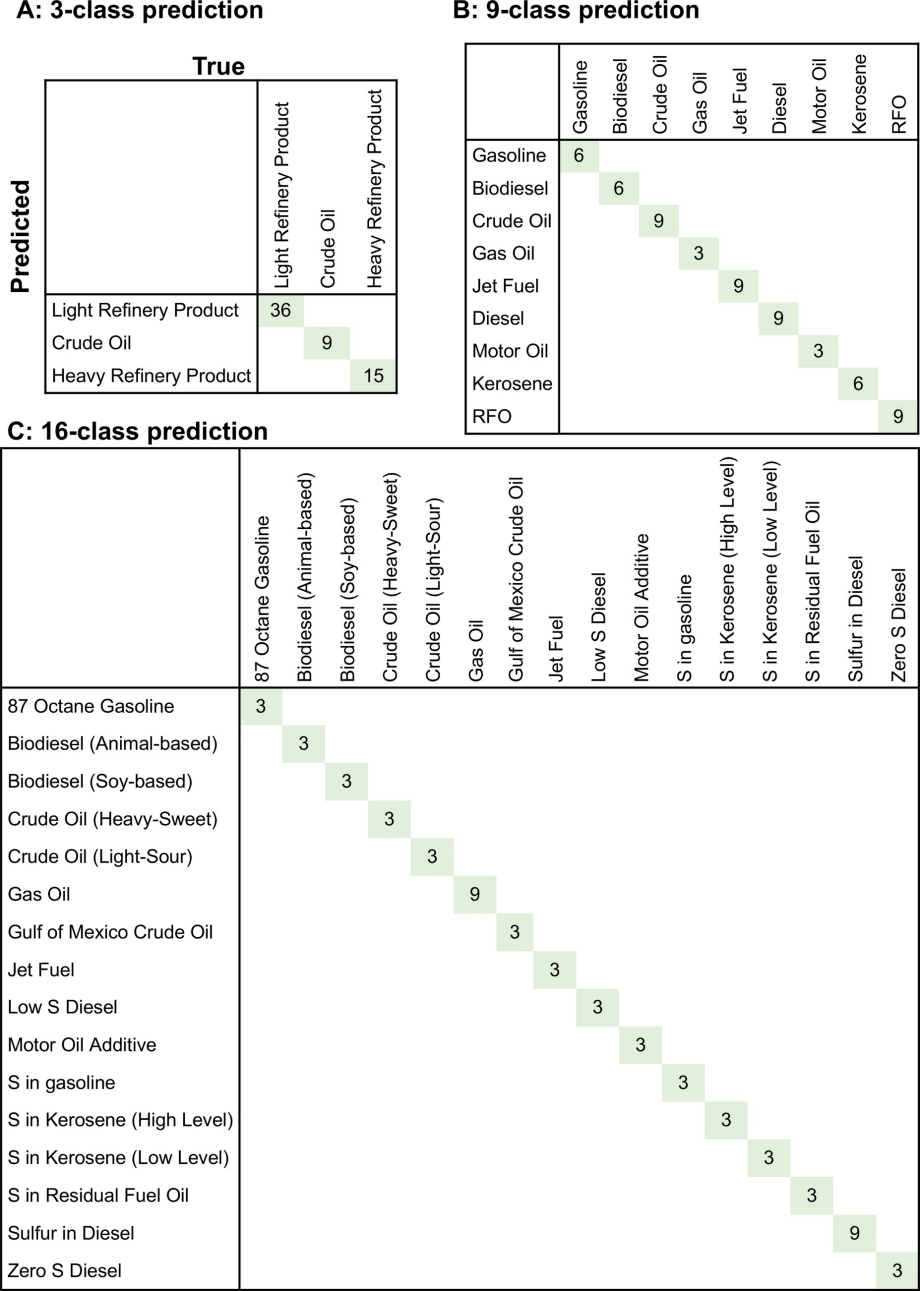
Confusion matrices for SRM sample classification with 3 replicates. (A) 3-class, (B) 9-class, and (C) 16-class grouping.

**Fig 6:**
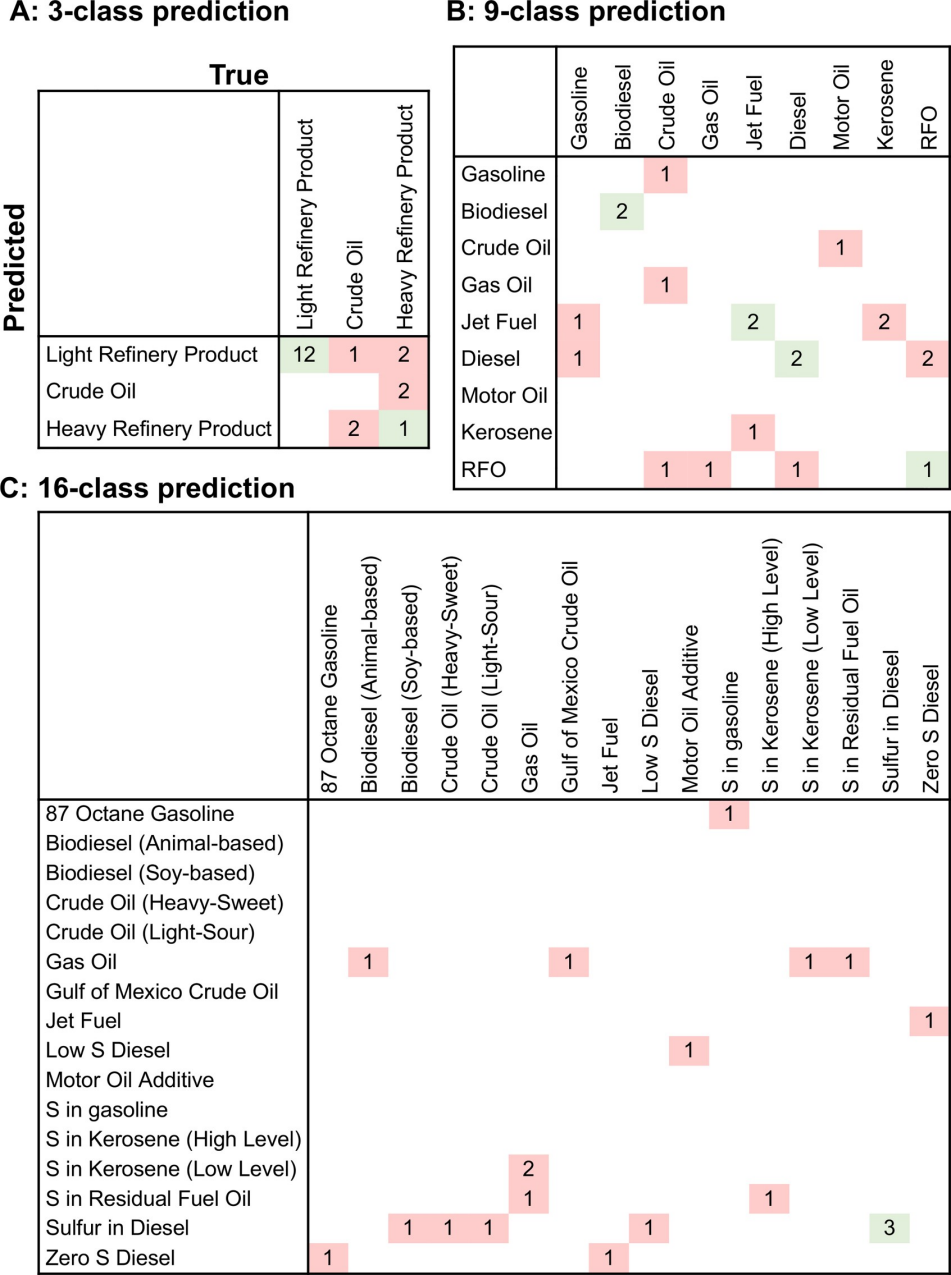
Confusion matrices for SRM sample classification with 1 replicate. (A) 3-class, (B) 9-class, and (C) 16-class grouping.

Figs 5 and 6 demonstrate the confusion matrices obtained from the trained Random Forest classifiers. These matrices report known (“true”) and predicted (through the trained Random Forest classifier) classes for each SRM sample. The results showed that we achieved 100% classification accuracy when we used all replicates provided in Table 1 (Table 3, Fig 5). The classification accuracy decreased to 65%, 35%, and 15% for 3, 9, and 16-class groupings when we only used 1 out 3 replicates (Table 3, Fig 6). The main reason for this fact is that the number of samples per group decreases as the number of classes increases. In particular, 14 out of 16 classes were represented with only a single sample during model training for the 16-class predictions (Fig 6C). Similarly, 5 out 9 classes were represented with only a single sample during model training for the 9-class predictions (Fig 6B). This decrease in the amount of information per class makes model learning significantly challenging (Table 3). Hence, we can conclude that single sample per class does not provide adequate information to capture the individual class characteristics. Moreover, the high-dimensional nature of the GC-MS data with 55 features further hindered the classification accuracy of SRM materials when using only one sample per category. Yet, the prediction accuracies of the classifiers for each analysis were higher than those for random prediction, indicating they were statistically significant (Table 3). This was validated through *p*-value calculations by using the original and 1000 random permutation grouping results (Table 3). The confusion matrices generated from the average of 1000 permutations of SRM samples are provided in S1 Fig and S2 Fig for 3 and 1 replicates, respectively.

**Table 3:** Classification accuracy of SRM samples using sample replicates.

Prediction class type	Number of sample replicates used	Classification accuracy	Classification accuracy (permuted)	*p-*value
3-class	3	100%	44.8±7.0%	0.000
	1	65%	48.0±9.2%	0.023
9-class	3	100%	10.9±5.1%	0.000
	1	35%	6.6±6.9%	0.000
16-class	3	100%	6.5±4.1%	0.000
	1	15%	4.2±5.1%	0.019

Similar trend was observed with Petroleum UVCB samples, where we built classification models using only 1 replicate of each sample (Table 4, S3 Fig). The results demonstrated that classification model accuracies for the Petroleum UVCB samples were not satisfactory, where the only statistically significant result was obtained from IM-MS data with 60% classification accuracy. Therefore, in order to build an accurate classification model, we need higher number of experimental replicates for each particular complex substance to capture and learn the nonlinear characteristics of their chemical complexity. Although clustering can group the samples accurately independent of the sample size, given that measurements are significantly distinct from each other, sufficient data sample size is essential during the classification model building. Nonetheless, each experimental replicate leads to an additional cost and requires extra time, and resources. Thus, minimizing the number of sample replicates while achieving accurate predictive classifiers is of utmost importance. In this work, we observed that, given high quality analytical chemistry data, 3 replicates were sufficient to build accurate and robust classifiers. It is important to note that the sample size is critical during the model training phase, where the models benefit from higher number of samples. However, this is not the case for the testing phase where a single experiment is sufficient to predict its class information of an unknown complex substance.

**Table 4:** Classification accuracy of Petroleum UVCB samples.

Prediction class type	Analytical technique used	Classification accuracy	Classification accuracy (permuted)	*p-*value
3-class (VHGO, SRGO, OGO)	GC-MS	40.0%	39.9±13.5%	0.395
	GC×GC-FID	46.7%	39.4±13.9%	0.222
	IM-MS	60.0%	41.4±12.0%	0.047

### 3.3. Importance of substance number to class ratio during supervised analysis

Another important question that needs to be answered is the following: How precisely can we categorize a new, unknown, substance with a data-driven model which is trained with an analytical chemistry profile database of categorized substances with no prior labeled samples of the tested substance? Hypothetically, one can accurately classify an unknown substance when provided a classifier trained with an analytical chemistry profile database that includes a high number of substances per class. In other words, a new substance can be precisely labeled if the analytical chemistry profile database provides an accurate mean profile of particular classes. Here, we used 20 SRM substances to understand whether the number of substance-to-class ratio of the data set can enable accurate categorization of each SRM substance. To this end, we developed one Random Forest classification model per each of the 20 SRM substances and reported the overall classification accuracy. In particular, we excluded the analytical chemistry profile information from all 3 replicates of the selected SRM substance during model training, and then predicted the category of with the trained model.

For 3-class predictions, the classification accuracy was obtained as 75% (Table 5), where the confusion matrices for original and average of 1000 permutations of SRM substance group labels are provided in S4 Fig. However, the calculated *p*-value (Table 5) showed that the developed data-driven models were statistically insignificant, which we attribute to a low number of substance-to-class ratio. This ratio deteriorates as the number of categorizes increase for 20 SRM substances. As a confirmation of our observation, we also ran a random forest model in which we calculated an average feature profile per substance, collapsing the replicates in to one artificial feature vector. The resulting classification accuracy was very similar (in the range of 70%-75%, not shown).

**Table 5:** 3-class classification accuracy of SRM substances excluding sample replicates.

Prediction class type	Classification accuracy	Classification accuracy (permuted)	*p-*value
3-class	75%	71.8±3.6%	0.092

S2 Table tabulates the number of substances and sample replicates per each class for 3-class, 9-class, and 16-class categorization. As can be seen from the S2 Table, removing all 3 replicates of a substance often corresponds to removing all samples of a class in several instances of a 9-class and 16-class analysis, thus hinders us to develop classification models for 2 (Category 4 and 7), and 14 categories (excluding only Category 6 and 15) for 9-class, and 16-class analysis, respectively. Therefore, we cannot report an overall classification accuracy for 9, and 16-class analysis. These results indicate that a high number for the substance-to-class ratio is crucial for accurate classification of an unknown substance with data-driven models that are trained without any previous samples of the tested substance. Therefore, we conclude that (i) continuous improvement of the analytical chemistry profile database used for model training with the addition of categorized substances per each class, and (ii) continuous update of the data-driven model are essential and necessary for accurate categorization of a new, unknown substance.

### 3.4. Facilitation of data interpretation via ToxPi representation

**Fig 7:**
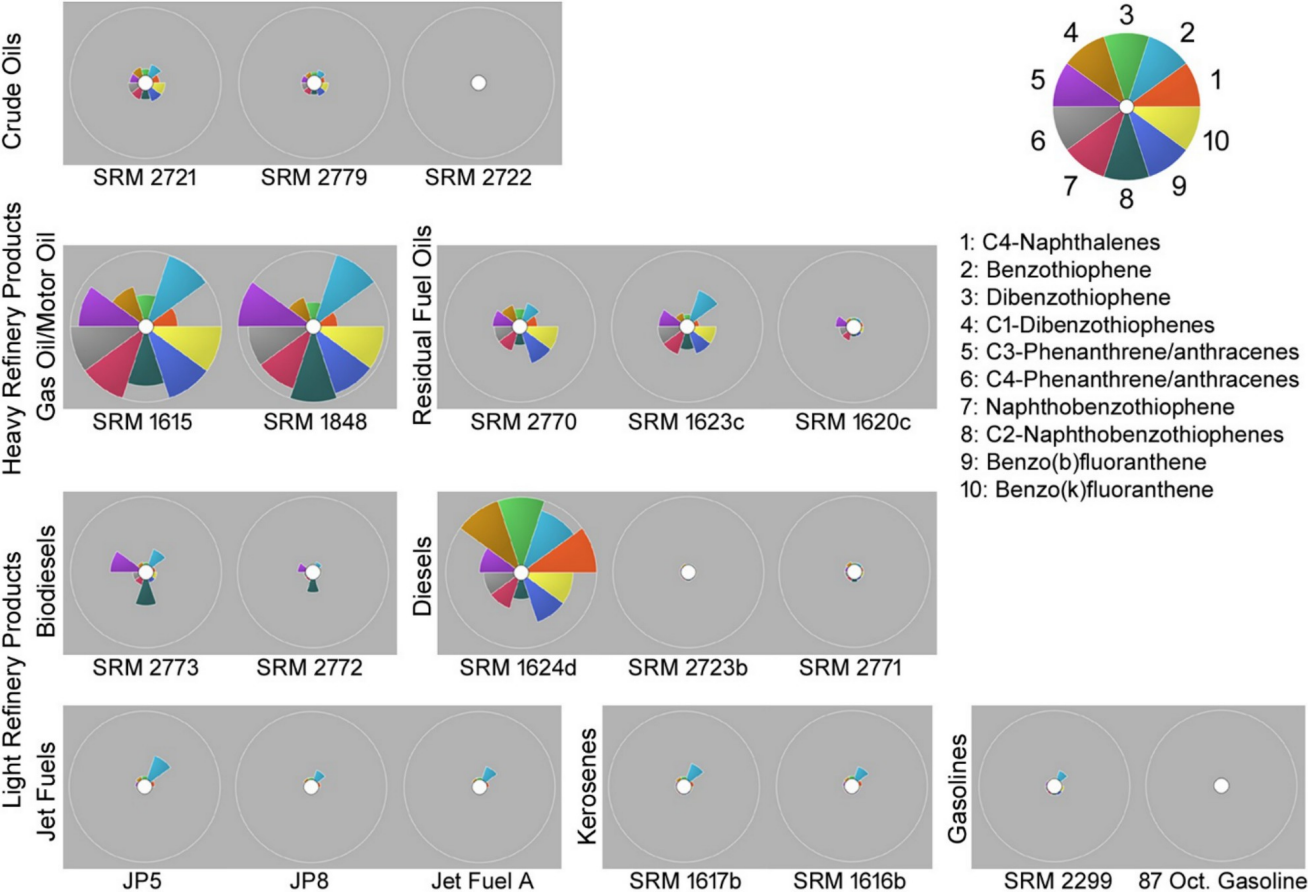
ToxPi visualization of SRM samples using top 10 most informative chromatographic features.

In addition to developing highly accurate classifier models to predict group/class information of an unknown complex substance using the sample replicates of categorized substances, we also reported the top 10 most informative features that distinctively identify the class patterns of SRM materials (Table 6). These informative features help us to facilitate the visual communication of the findings via ToxPi visualization as shown in Fig 7.

**Table 6:** Top 10 most informative GC-MS chromatographic features with respect to the classification accuracy of the petroleum substances. See S3–S5 Tables for the list of all chromatographic features and their respective ranks in each analysis.

	3-Class prediction		9-Class prediction		16-Class prediction	
GC-MS Chromatographic Feature	Rank*	Mean decrease in accuracy (%)^#^	Rank	Mean decrease in accuracy (%)	Rank	Mean decrease in accuracy (%)
C4-Naphthalenes	6	6.82	13	8.68	1	10.38
Naphthobenzothiophene	10	6.54	14	8.56	7	9.44
C2-Naphthobenzothiophenes	24	6.12	2	9.09	10	9.40
Benzothiophene	21	6.25	3	9.08	14	9.25
C3-Phenanthrene/anthracenes	5	6.90	6	8.86	33	8.55
C4-Phenanthrene/anthracenes	8	6.58	35	7.76	3	9.57
Benzo(b)fluoranthene	19	6.28	20	8.42	8	9.42
Dibenzothiophene	41	5.76	4	9.02	4	9.56
C1-Dibenzothiophenes	2	7.10	22	8.35	26	8.69
Benzo(k)fluoranthene	27	6.06	16	8.53	11	9.34

*Rank of the feature among 55 total for each classification analysis (3-, 9-, or 16-class prediction). Top 10 features with the overall highest rank in all three analyses were selected.

^#^Mean decrease in the accuracy of classification when this feature is removed from the analysis.

**Fig 8:**
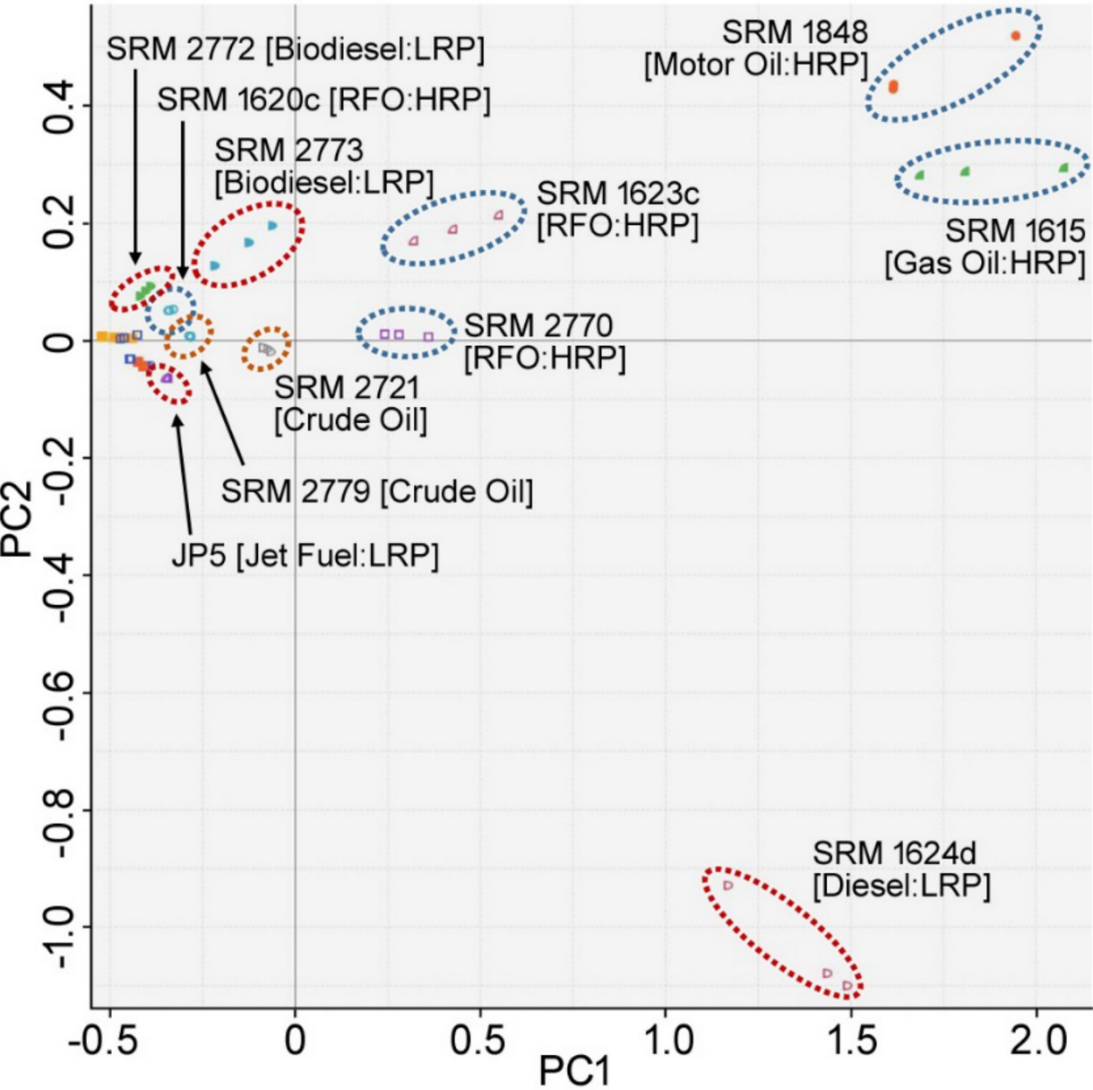
PCA of ToxPi scores.

The ToxPi profiles of SRM samples successfully demonstrated the distinct nature of gas/motor oils, biodiesels, and crude oils (with the exception of SRM 2722) with respect to the rest of SRMs. Specifically for crude oils, all of the top 10 chromatographic features helped to identify crude oils among SRMs. Whereas for gas/motor oils, the profiles revealed the importance of C2-napthobenzothiophenes for further identification between them. Moreover, the ToxPi profiles showed that C3-phenanthrene/anthracenes, C2-naphthobenzothiophenes, and benzothiophene measurements were the characteristics of biodiesel samples and can differentiate them from the rest of the SRMs. We also observed the high similarity among a subgroup of light refinery products that includes jet fuels, kerosenes and gasolines, where the weight of benzothiophene remained to be the unique characteristic among all of them. This proves that the GC-MS data could not provide clear distinction among these substances. Finally, we noted the major difference between diesel samples. Unlike the other two diesel samples, SRM 2723b and SRM 2771, most of the top 10 selected analytical features were significant for identifying SRM 1624d. In particular, dibenzothiophene, C1-dibenzothiophenes and C4-naphthalenes were the distinct measurements that differentiate SRM 1624d from the rest of the SRMs the most. Furthermore, the PCA of the extracted ToxPi scores helps us to depict the distinction between the complex substances by using the most informative analytical feature information (Fig 8).

### 3.5. Comparison of GC×GC-FID, GC-MS, and IM-MS techniques via Fowlkes-Mallows index

In addition to quantifying the grouping quality and class information, it is imperative to investigate the appropriate analytical chemistry technique that produces the optimal grouping for substances with complex chemistries. The majority of regulatory and standardized chemical compositional analysis protocols utilize GC-MS as the instrument of choice to fingerprint UVCB substances. Generally, a GC-MS instrument employs a capillary column, heated by an oven at a predetermined temperature gradient in order to separate compounds by boiling point and polarity. The eluting compounds are then ionized and analyzed by a detector. Since molecules of specific molecular classes maintain distinct mass ion fracture patterns, GC-MS is able to differentiate ion signals from multiple compounds. However, the column peak capacity of a GC-MS can become overloaded, causing a baseline hump termed as an unresolved complex mixture (UCM). In such cases, the column no longer has the resolving power to separate all the compounds within the sample, which is typically observed in petroleum substance analysis, since an individual petroleum substance contains more than 10,000 different chemical compounds. This may limit the number of molecules that can be effectively differentiated by the instrument and hinder a robust chemical fingerprint production.

However, in recent years, instrument resolution power and sensitivity has increased, allowing for more detailed characterization of complex substances. The incorporation of two gas chromatography columns with different selectivity (GC×GC-FID) increases the peak capacity of the instrument and allows for improved separation of molecules that form a UCM under GC-MS analysis. Moreover, ion mobility mass spectrometry (IM-MS) incorporates unique ionization methods, electron spray (ESI) or atmospheric photo ionization (APPI), along with separation techniques based on size, shape, and charge of the ionized molecule. This further increases the analytical sensitivity and enables improved chemical fingerprinting. Although these two techniques further enhance the ability to characterize complex substances like petroleum products, their application is still novel and not widely studied within the scientific, regulatory, or industrial communities [14, 47]. Despite the technological advances that are introduced by GC×GC-FID, and IM-MS techniques over GC-MS, there is no evidence examining any potential improvements on complex substance grouping. Therefore, we utilized the Fowlkes-Mallows index to provide comparative assessment between these three analytical chemistry techniques using the Petroleum UVCB samples.

**Fig 9:**
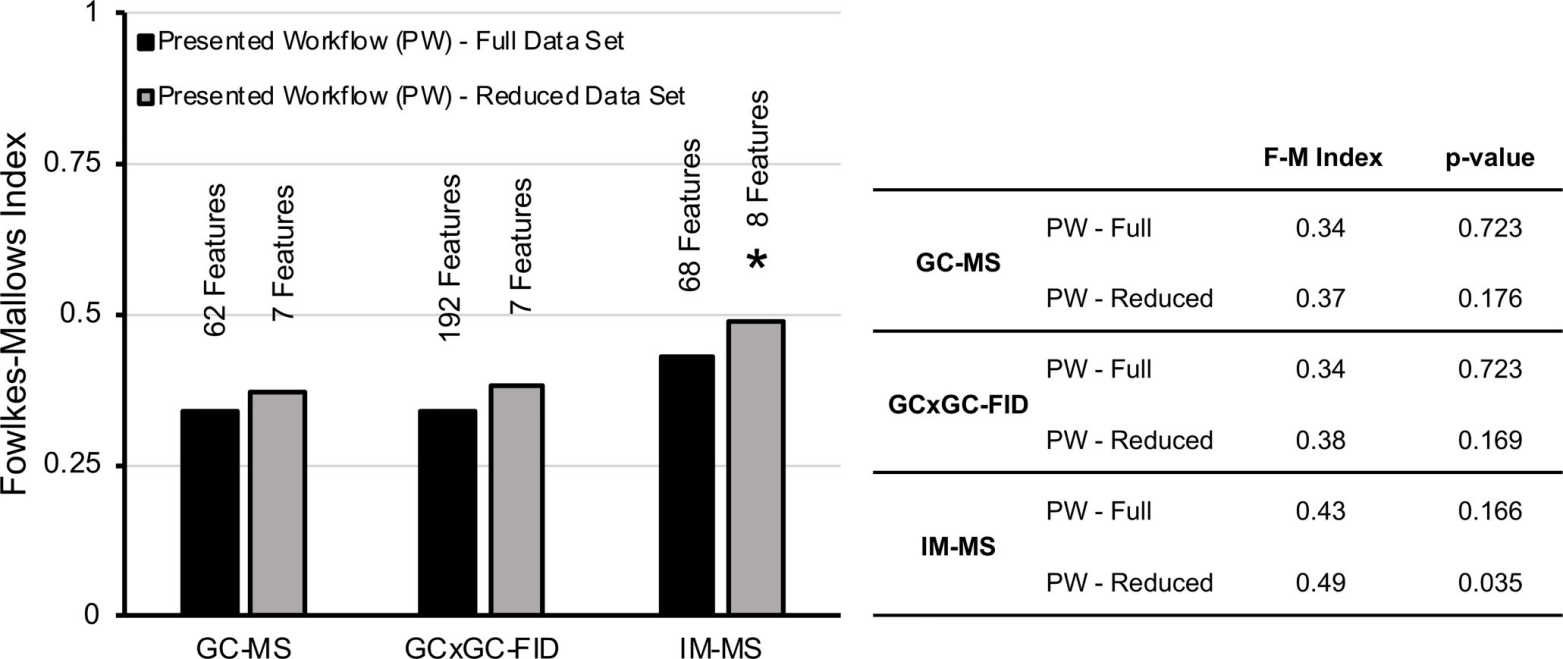
F-M index for the outcomes of clustering of Petroleum UVCB samples analyzed using 3 different techniques. * indicates that the results are statistically significant.

**Fig 10:**
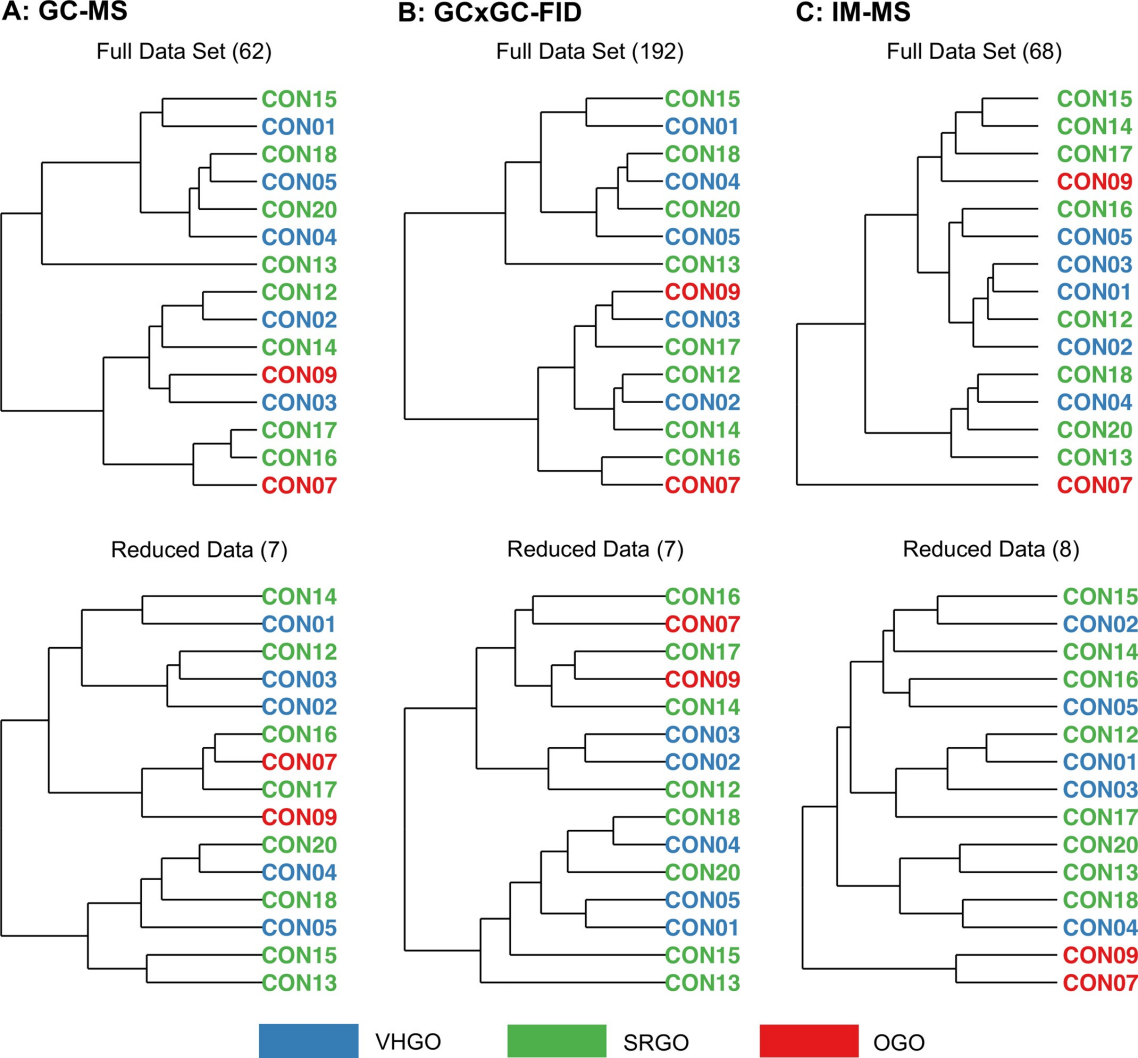
Dendrograms for Petroleum UVCB samples clustering from the reduced data set analyzed using 3 different techniques.

Fig 9 demonstrates that GC×GC-FID and GC-MS yielded statistically insignificant F-M indices due to the limited sample size. IM-MS was the only one yielding statistically significant results, only after dimensionality reduction, which provided the most useful information to reveal the class differences among the Petroleum UVCB samples. Their corresponding clustering dendrograms are provided in Fig 10. Specifically, the F-M index of the grouping of Petroleum UVCB samples with 8 features generated via the IM-MS technique was 0.49. Although we could not draw specific conclusions among GC×GC-FID and GC-MS, we can report that IM-MS performed superior than the other two techniques in terms of capturing the chemical characteristics of complex substances.

## 4. Conclusion

In this study, we established a data-driven framework for optimal grouping complex chemical substances based on their chemical characteristics, and provided quantitative and visual evaluation to facilitate the interpretation of the complex chemical nature of substances/mixtures. The designed framework consists of two analysis workflows with two different perspectives. In unsupervised analysis workflow, we examined the grouping of the complex substances by using their chemical fingerprints derived from various analytical techniques, and quantitatively compared the grouping hierarchy to a reference categorization through F-M index. In contrast, in a supervised analysis workflow, we benefited from the “read-across” hypothesis, that is similar complex substances that are grouped together based on their chemical structure (i.e. manufacturing category) are prone to behave similarly in terms of environmental health risk assessment. Hence, we can train highly accurate classification models by using the available information on categorization of known complex substances. The generated models can then be used to predict the environmental health impact of future unknown complex substances. The common highlight of both workflows was on the quantitative metrics, which immensely facilitated the comparative assessment of different parameters, such as distinct analytical techniques, data set sizes, or different number of categorization of samples to elucidate the optimal grouping of complex substances. Additionally, we incorporated the ToxPi representation of complex substances with the most informative analytical features to further deliver insights from the developed data-driven classification models.

Our results have shown that in order to assess the statistical significance of grouping results, it is highly important to permute category labels of complex substances and to calculate *p-*value for the obtained results regardless of the selected workflow. In addition, the dimensionality reduction played a key role in reducing the noise in the extracted high-dimensional analytical chemistry profiles. Dimensionality reduction allowed similar or higher grouping quality with significantly reduced number of measurements. The selection of the most informative features further improved data interpretation significantly through advanced data visualization techniques, such as the ToxPi representation. This further facilitated the communication of complex substance characteristics with regulatory decision-makers.

## Supporting information

S1 Text Data source and R Markdown documentation. (DOCX)

S1 Table List of selected analytes from the GC-MS data of SRM samples for grouping analysis. (DOCX)

S2 Table Number of SRM substances and samples for 3-class, 9-class, and 16-class categorizations. (DOCX)

S3 Table List of all chromatographic features and their respective ranks in 3 class grouping analysis. (DOCX)

S4 Table List of all chromatographic features and their respective ranks in 9 class grouping analysis. (DOCX)

S5 Table List of all chromatographic features and their respective ranks in 16 class grouping analysis. (DOCX)

S1 Fig Average confusion matrices of 1000 permutations for SRM sample classification. (A) 3-class grouping, (B) 9-class grouping, and (C) 16-class grouping with 3 replicates.
(DOCX)

S2 Fig Average confusion matrices of 1000 permutations for SRM sample classification. (A) 3-class grouping, (B) 9-class grouping, and (C) 16-class grouping with 1 replicate.
(DOCX)

S3 Fig Original and average confusion matrices of 1000 permutations for Petroleum UVCB sample classification. (A) GC-MS, (B) GC×GC-FID, and (C) IM-MS data.
(DOCX)

S4 Fig Original and average confusion matrices of 1000 permutations for SRM substance classification. (DOCX)
