# Supplementary material for: Grouping of complex substances using analytical chemistry data: A framework for quantitative evaluation and visualization
Source: PLoS One. 2019 Oct 10;14(10):e0223517. doi: 10.1371/journal.pone.0223517 (PMC6786635; doi:10.1371/journal.pone.0223517)
Supplement: S1 Text — (DOCX) [file pone.0223517.s001.docx]

**Supplementary Material for Grouping of Complex Substances Using Analytical Chemistry Data: A Framework for Quantitative Evaluation and Visualization**

Melis Onel^1,2^, Burcu Beykal^1,2^, Kyle Ferguson^3^, Weihsueh A. Chiu^3^, Thomas J. McDonald^4^,

Lan Zhou^5^, John S. House^6^, Fred A. Wright^6,7^, David A. Sheen^8^,

Ivan Rusyn^3,&*^, Efstratios N. Pistikopoulos^1,2,&*^

^1^Artie McFerrin Department of Chemical Engineering, Texas A&M University, College Station, TX 77843, United States of America

^2^Texas A&M Energy Institute, Texas A&M University, College Station, TX, United States of America

^3^Department of Veterinary Integrative Biosciences, Texas A&M University, College Station, TX, United States of America

^4^Department of Environmental and Occupational Health, Texas A&M University, College Station, TX, United States of America

^5^Department of Statistics, Texas A&M University, College Station, TX, United States of America

^6^Bioinformatics Research Center, North Carolina State University, Raleigh, NC, United States of America

^7^Departments of Statistics and Biological Sciences, North Carolina State University, Raleigh, NC

^8^Chemical Sciences Division, National Institute of Standards and Technology, Gaithersburg, MD, United States of America

& These authors contributing equally to this work.

*corresponding authors

[irusyn@cvm.tamu.edu](mailto:irusyn@cvm.tamu.edu)

[stratos@tamu.edu](mailto:stratos@tamu.edu)

The R Markdown documentation of unsupervised and supervised analysis workflow can be accessed through <http://parametric.tamu.edu/research/Onel_etAl_2019_Rmarkdown.html>

The GC-MS, GCxGC-FID, and IM-MS data of Petroleum UVCB, and GC-MS data of SRM samples can be downloaded from http://paroc.tamu.edu/Software/Onel_etAl_2019_data.zip
