# Supplementary material for: Grouping of complex substances using analytical chemistry data: A framework for quantitative evaluation and visualization
Source: PLoS One. 2019 Oct 10;14(10):e0223517. doi: 10.1371/journal.pone.0223517 (PMC6786635; doi:10.1371/journal.pone.0223517)
Supplement: S1 Table — (DOCX) [file pone.0223517.s002.docx]

**S1 Table. List of selected analytes from the GC-MS data of SRM samples for grouping analysis.**

| Selected Analytes | Quantitation Ion |
| --- | --- |
| Decalin | 138 |
| C1-decalins | 152 |
| C2-decalins | 166 |
| C3-decalins | 180 |
| Naphthalene | 128 |
| C1-naphthalenes | 142 |
| C2-naphthalenes | 156 |
| C3-naphthalenes | 170 |
| C4-naphthalenes | 184 |
| Benzothiophene | 134 |
| C1-benzothiophenes | 148 |
| C2-benzothiophenes | 162 |
| C3-benzothiophenes | 176 |
| Biphenyl | 154 |
| Acenaphthylene | 152 |
| Acenaphthene | 154 |
| Dibenzofuran | 168 |
| Fluorene | 166 |
| C1-fluorenes | 180 |
| C2-fluorenes | 194 |
| C3-fluorenes | 208 |
| Dibenzothiophene | 184 |
| C1-dibenzothiophenes | 198 |
| C2-dibenzothiophenes | 212 |
| C3-dibenzothiophenes | 226 |
| C4-dibenzothiophenes | 240 |
| Phenanthrene | 178 |
| Anthracene | 178 |
| C1-phenanthrene/anthracenes | 192 |
| C2-phenanthrene/anthracenes | 206 |
| C3-phenanthrene/anthracenes | 220 |
| C4-phenanthrene/anthracenes | 234 |
| Naphthobenzothiophene | 234 |
| C1-naphthobenzothiophenes | 248 |
| C2-naphthobenzothiophenes | 262 |
| C3-naphthobenzothiophenes | 276 |
| Fluoranthene | 202 |
| Pyrene | 202 |
| C1-fluoranthene/pyrenes | 216 |
| C2-fluoranthene/pyrenes | 230 |
| C3-fluoranthene/pyrenes | 244 |
| Benz(a)anthracene | 228 |
| Chrysene | 228 |
| C1-chrysenes/benzo(a)anthracenes | 242 |
| C2-chrysenes/benzo(a)anthracenes | 256 |
| C3-chrysenes/benzo(a)anthracenes | 270 |
| C4-chrysenes/benzo(a)anthracenes | 284 |
| Benzo(b)fluoranthene | 252 |
| Benzo(k)fluoranthene | 252 |
| Benzo(e)pyrene | 252 |
| Benzo(a)pyrene | 252 |
| Indeno(1,2,3-cd)pyrene | 276 |
| Dibenzo(a,h)anthracene | 278 |
| Benzo(g,h,i)perylene | 276 |
| Perylene | 252 |
