# Supplementary material for: Grouping of complex substances using analytical chemistry data: A framework for quantitative evaluation and visualization
Source: PLoS One. 2019 Oct 10;14(10):e0223517. doi: 10.1371/journal.pone.0223517 (PMC6786635; doi:10.1371/journal.pone.0223517)
Supplement: S2 Table — (DOCX) [file pone.0223517.s003.docx]

**S2 Table. Number of SRM substances and samples for 3-class, 9-class, and 16-class categorizations.**

| Prediction class type | Class | Number of SRM substances | Number of classified samples |
| --- | --- | --- | --- |
| 3-class | 1 | 12 | 36 |
|  | 2 | 3 | 9 |
|  | 3 | 5 | 15 |
| 9-class | 1 | 2 | 6 |
|  | 2 | 2 | 6 |
|  | 3 | 3 | 9 |
|  | 4 | 1 | 3 |
|  | 5 | 3 | 9 |
|  | 6 | 3 | 9 |
|  | 7 | 1 | 3 |
|  | 8 | 2 | 6 |
|  | 9 | 3 | 9 |
| 16-class | 1 | 1 | 3 |
|  | 2 | 1 | 3 |
|  | 3 | 1 | 3 |
|  | 4 | 1 | 3 |
|  | 5 | 1 | 3 |
|  | 6 | 3 | 9 |
|  | 7 | 1 | 3 |
|  | 8 | 1 | 3 |
|  | 9 | 1 | 3 |
|  | 10 | 1 | 3 |
|  | 11 | 1 | 3 |
|  | 12 | 1 | 3 |
|  | 13 | 1 | 3 |
|  | 14 | 1 | 3 |
|  | 15 | 3 | 9 |
|  | 16 | 1 | 3 |
