# Supplementary material for: Grouping of complex substances using analytical chemistry data: A framework for quantitative evaluation and visualization
Source: PLoS One. 2019 Oct 10;14(10):e0223517. doi: 10.1371/journal.pone.0223517 (PMC6786635; doi:10.1371/journal.pone.0223517)
Supplement: S3 Table — (DOCX) [file pone.0223517.s004.docx]

**S3 Table. List of all chromatographic features and their respective ranks in 3 class grouping analysis.**

| Rank | Features | Mean Decrease in Accuracy (%) |
| --- | --- | --- |
| 1 | C2-ChrysenesBenzoaanthracenes | 7.521 |
| 2 | C1-Dibenzothiophenes | 7.100 |
| 3 | Dibenzofuran | 7.080 |
| 4 | C2-Dibenzothiophenes | 6.910 |
| 5 | C3-Phenanthreneanthracenes | 6.902 |
| 6 | C4-Naphthalenes | 6.821 |
| 7 | C2-Fluoranthenepyrenes | 6.696 |
| 8 | C4-Phenanthreneanthracenes | 6.575 |
| 9 | C2-Decalins | 6.571 |
| 10 | Naphthobenzothiophene | 6.540 |
| 11 | C4-Dibenzothiophenes | 6.490 |
| 12 | C1-Benzothiophenes | 6.489 |
| 13 | C3-Dibenzothiophenes | 6.468 |
| 14 | Fluoranthene | 6.456 |
| 15 | Acenaphthene | 6.453 |
| 16 | C3-Fluorenes | 6.311 |
| 17 | Acenaphthylene | 6.302 |
| 18 | C3-Naphthalenes | 6.300 |
| 19 | Benzobfluoranthene | 6.279 |
| 20 | C2-Phenanthreneanthracenes | 6.266 |
| 21 | Benzothiophene | 6.248 |
| 22 | Pyrene | 6.199 |
| 23 | C2-Fluorenes | 6.142 |
| 24 | C2-Naphthobenzothiophenes | 6.121 |
| 25 | Naphthalene | 6.100 |
| 26 | C1-Decalins | 6.092 |
| 27 | Benzokfluoranthene | 6.064 |
| 28 | C2-Naphthalenes | 6.021 |
| 29 | Benzoghiperylene | 5.993 |
| 30 | C1-Fluoranthenepyrenes | 5.950 |
| 31 | Indeno123cdpyrene | 5.932 |
| 32 | Anthracene | 5.907 |
| 33 | C1-Phenanthreneanthracenes | 5.873 |
| 34 | Decalin | 5.862 |
| 35 | Biphenyl | 5.848 |
| 36 | Benzoepyrene | 5.846 |
| 37 | Benzaanthracene | 5.808 |
| 38 | Dibenzoahanthracene | 5.798 |
| 39 | Phenanthrene | 5.791 |
| 40 | C4-ChrysenesBenzoaanthracenes | 5.761 |
| 41 | Dibenzothiophene | 5.760 |
| 42 | C1-Naphthalenes | 5.593 |
| 43 | C1-Naphthobenzothiophenes | 5.400 |
| 44 | Chrysene | 5.277 |
| 45 | C3-Benzothiophenes | 5.274 |
| 46 | C3-Decalins | 5.270 |
| 47 | Benzoapyrene | 5.248 |
| 48 | Perylene | 5.238 |
| 49 | Fluorene | 5.150 |
| 50 | C1-Fluorenes | 5.127 |
| 51 | C1-ChrysenesBenzoaanthracenes | 5.071 |
| 52 | C3-Naphthobenzothiophenes | 4.904 |
| 53 | C3-ChrysenesBenzoaanthracenes | 4.861 |
| 54 | C3-Fluoranthenepyrenes | 4.809 |
| 55 | C2-Benzothiophenes | 4.767 |
