# Supplementary material for: Grouping of complex substances using analytical chemistry data: A framework for quantitative evaluation and visualization
Source: PLoS One. 2019 Oct 10;14(10):e0223517. doi: 10.1371/journal.pone.0223517 (PMC6786635; doi:10.1371/journal.pone.0223517)
Supplement: S4 Table — (DOCX) [file pone.0223517.s005.docx]

**S4 Table. List of all chromatographic features and their respective ranks in 9 class grouping analysis.**

| Rank | Features | Mean Decrease in Accuracy (%) |
| --- | --- | --- |
| 1 | Fluorene | 9.152 |
| 2 | C2-Naphthobenzothiophenes | 9.089 |
| 3 | Benzothiophene | 9.083 |
| 4 | Dibenzothiophene | 9.018 |
| 5 | C3-ChrysenesBenzoaanthracenes | 8.970 |
| 6 | C3-Phenanthreneanthracenes | 8.855 |
| 7 | Decalin | 8.782 |
| 8 | Benzoapyrene | 8.781 |
| 9 | Anthracene | 8.738 |
| 10 | Benzoghiperylene | 8.727 |
| 11 | C2-Benzothiophenes | 8.723 |
| 12 | Naphthalene | 8.706 |
| 13 | C4-Naphthalenes | 8.677 |
| 14 | Naphthobenzothiophene | 8.564 |
| 15 | Phenanthrene | 8.562 |
| 16 | Benzokfluoranthene | 8.533 |
| 17 | C3-Naphthobenzothiophenes | 8.463 |
| 18 | Chrysene | 8.458 |
| 19 | C1-Phenanthreneanthracenes | 8.452 |
| 20 | Benzobfluoranthene | 8.420 |
| 21 | Fluoranthene | 8.367 |
| 22 | C1-Dibenzothiophenes | 8.350 |
| 23 | C2-Naphthalenes | 8.348 |
| 24 | C2-Fluorenes | 8.320 |
| 25 | C4-ChrysenesBenzoaanthracenes | 8.315 |
| 26 | C3-Benzothiophenes | 8.216 |
| 27 | Acenaphthylene | 8.074 |
| 28 | Benzaanthracene | 8.056 |
| 29 | Dibenzofuran | 8.013 |
| 30 | C1-Fluorenes | 7.999 |
| 31 | C1-Naphthalenes | 7.890 |
| 32 | C2-Fluoranthenepyrenes | 7.812 |
| 33 | Dibenzoahanthracene | 7.807 |
| 34 | C4-Dibenzothiophenes | 7.782 |
| 35 | C4-Phenanthreneanthracenes | 7.761 |
| 36 | Biphenyl | 7.719 |
| 37 | C2-Decalins | 7.682 |
| 38 | C3-Decalins | 7.679 |
| 39 | C3-Dibenzothiophenes | 7.607 |
| 40 | C1-Fluoranthenepyrenes | 7.580 |
| 41 | Perylene | 7.477 |
| 42 | C1-Naphthobenzothiophenes | 7.449 |
| 43 | Acenaphthene | 7.369 |
| 44 | C3-Naphthalenes | 7.340 |
| 45 | C1-Decalins | 7.332 |
| 46 | C2-ChrysenesBenzoaanthracenes | 7.292 |
| 47 | Indeno123cdpyrene | 7.280 |
| 48 | Benzoepyrene | 7.226 |
| 49 | C1-ChrysenesBenzoaanthracenes | 7.226 |
| 50 | C2-Dibenzothiophenes | 7.170 |
| 51 | C1-Benzothiophenes | 7.133 |
| 52 | C3-Fluorenes | 7.123 |
| 53 | C3-Fluoranthenepyrenes | 7.113 |
| 54 | C2-Phenanthreneanthracenes | 6.985 |
| 55 | Pyrene | 6.700 |
