# Supplementary material for: Grouping of complex substances using analytical chemistry data: A framework for quantitative evaluation and visualization
Source: PLoS One. 2019 Oct 10;14(10):e0223517. doi: 10.1371/journal.pone.0223517 (PMC6786635; doi:10.1371/journal.pone.0223517)
Supplement: S5 Table — (DOCX) [file pone.0223517.s006.docx]

**S5 Table. List of all chromatographic features and their respective ranks in 16 class grouping analysis.**

| Rank | Features | Mean Decrease in Accuracy (%) |
| --- | --- | --- |
| 1 | C4-Naphthalenes | 10.376 |
| 2 | C1-Fluorenes | 9.572 |
| 3 | C4-Phenanthreneanthracenes | 9.567 |
| 4 | Dibenzothiophene | 9.556 |
| 5 | C1-Fluoranthenepyrenes | 9.537 |
| 6 | Benzoepyrene | 9.447 |
| 7 | Naphthobenzothiophene | 9.442 |
| 8 | Benzobfluoranthene | 9.424 |
| 9 | Benzoapyrene | 9.422 |
| 10 | C2-Naphthobenzothiophenes | 9.398 |
| 11 | Benzokfluoranthene | 9.341 |
| 12 | C1-Naphthobenzothiophenes | 9.329 |
| 13 | Pyrene | 9.313 |
| 14 | Benzothiophene | 9.254 |
| 15 | C1-Decalins | 9.179 |
| 16 | C2-Fluorenes | 9.168 |
| 17 | C2-Naphthalenes | 9.121 |
| 18 | C3-Naphthalenes | 9.041 |
| 19 | C1-Naphthalenes | 8.956 |
| 20 | C1-ChrysenesBenzoaanthracenes | 8.856 |
| 21 | Fluoranthene | 8.844 |
| 22 | Benzaanthracene | 8.829 |
| 23 | C1-Phenanthreneanthracenes | 8.824 |
| 24 | C3-Fluoranthenepyrenes | 8.778 |
| 25 | C3-ChrysenesBenzoaanthracenes | 8.777 |
| 26 | C1-Dibenzothiophenes | 8.694 |
| 27 | Chrysene | 8.686 |
| 28 | Decalin | 8.667 |
| 29 | Acenaphthylene | 8.651 |
| 30 | Perylene | 8.619 |
| 31 | Acenaphthene | 8.588 |
| 32 | C2-Benzothiophenes | 8.563 |
| 33 | C3-Phenanthreneanthracenes | 8.549 |
| 34 | Fluorene | 8.515 |
| 35 | C3-Benzothiophenes | 8.514 |
| 36 | C2-Fluoranthenepyrenes | 8.503 |
| 37 | C4-Dibenzothiophenes | 8.503 |
| 38 | Dibenzofuran | 8.493 |
| 39 | C2-ChrysenesBenzoaanthracenes | 8.482 |
| 40 | C2-Decalins | 8.371 |
| 41 | Naphthalene | 8.343 |
| 42 | C3-Fluorenes | 8.335 |
| 43 | Biphenyl | 8.331 |
| 44 | Indeno123cdpyrene | 8.323 |
| 45 | C4-ChrysenesBenzoaanthracenes | 8.264 |
| 46 | C3-Dibenzothiophenes | 8.218 |
| 47 | Phenanthrene | 8.148 |
| 48 | Anthracene | 8.125 |
| 49 | Benzoghiperylene | 8.083 |
| 50 | C1-Benzothiophenes | 8.030 |
| 51 | C3-Naphthobenzothiophenes | 8.021 |
| 52 | C2-Dibenzothiophenes | 8.011 |
| 53 | C2-Phenanthreneanthracenes | 7.874 |
| 54 | Dibenzoahanthracene | 7.862 |
| 55 | C3-Decalins | 7.599 |

.
