# Supplementary material for: Grouping of complex substances using analytical chemistry data: A framework for quantitative evaluation and visualization
Source: PLoS One. 2019 Oct 10;14(10):e0223517. doi: 10.1371/journal.pone.0223517 (PMC6786635; doi:10.1371/journal.pone.0223517)
Supplement: S2 Fig — (A) 3-class grouping, (B) 9-class grouping, and (C) 16-class grouping with 1 replicate. (DOCX) [file pone.0223517.s008.docx]

**S2 Fig. Average confusion matrices of 1000 permutations for SRM sample classification.**

(A) 3-class grouping, (B) 9-class grouping, and (C) 16-class grouping with 1 replicate.

**
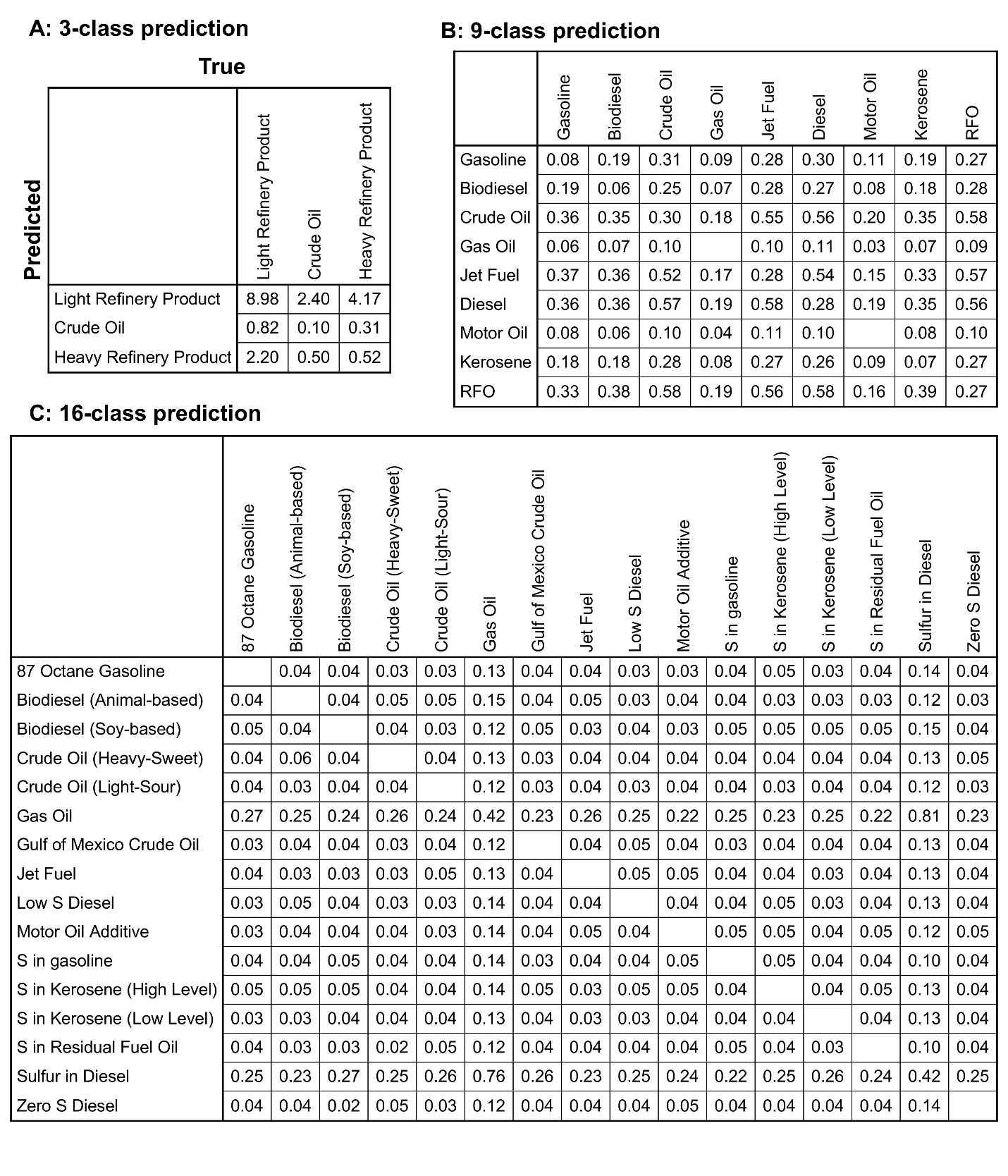
**
