# Supplementary material for: Grouping of complex substances using analytical chemistry data: A framework for quantitative evaluation and visualization
Source: PLoS One. 2019 Oct 10;14(10):e0223517. doi: 10.1371/journal.pone.0223517 (PMC6786635; doi:10.1371/journal.pone.0223517)
Supplement: S3 Fig — (A) GC-MS, (B) GC×GC-FID, and (C) IM-MS data. (DOCX) [file pone.0223517.s009.docx]

**S3 Fig. Original and average confusion matrices of 1000 permutations for Petroleum UVCB sample classification.**

(A) GC-MS, (B) GCxGC-FID, and (C) IM-MS data.

**
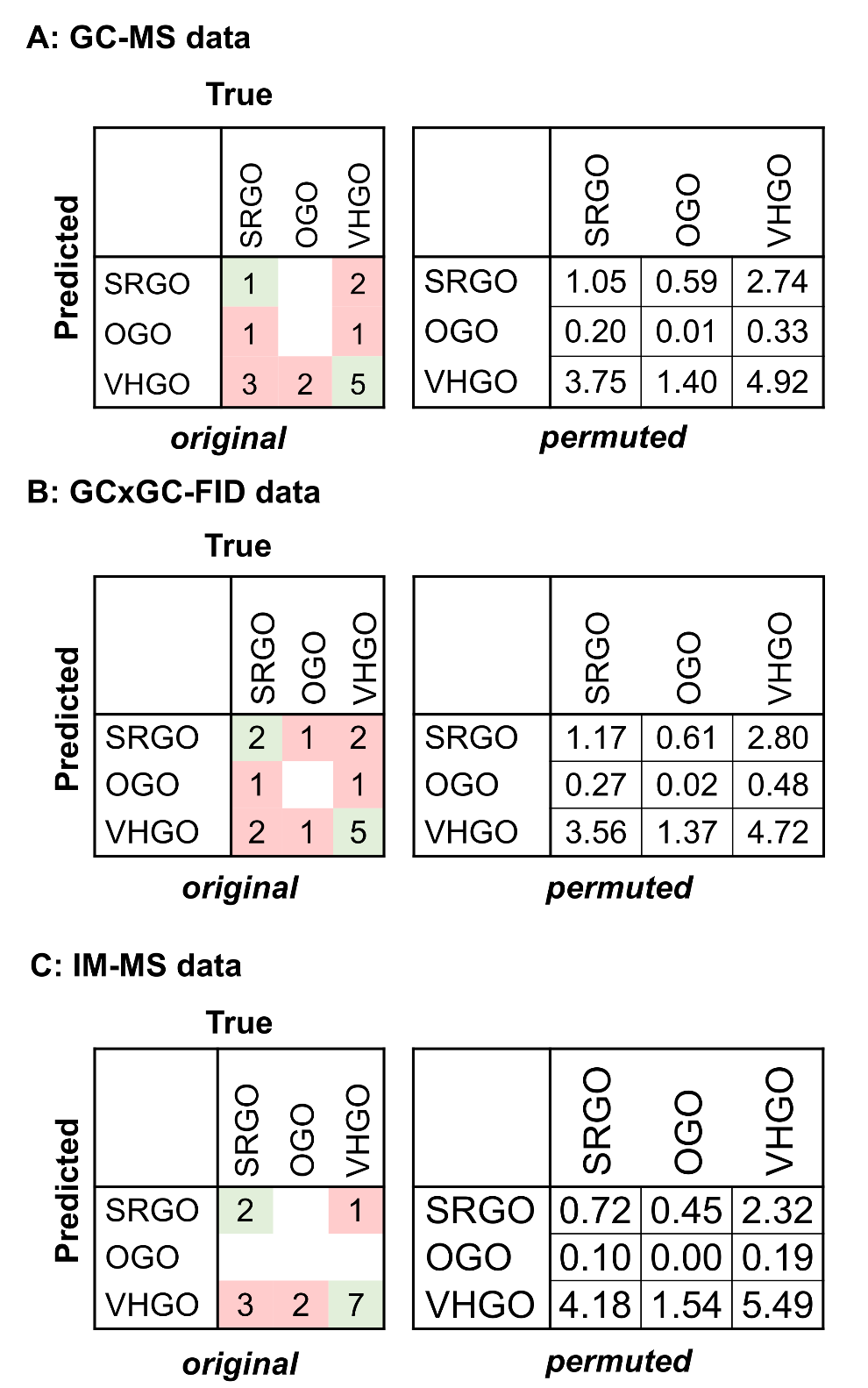
**
