# Supplementary material for: Grouping of complex substances using analytical chemistry data: A framework for quantitative evaluation and visualization
Source: PLoS One. 2019 Oct 10;14(10):e0223517. doi: 10.1371/journal.pone.0223517 (PMC6786635; doi:10.1371/journal.pone.0223517)

**S4 Fig. Original and average confusion matrices of 1000 permutations for SRM substance classification.**


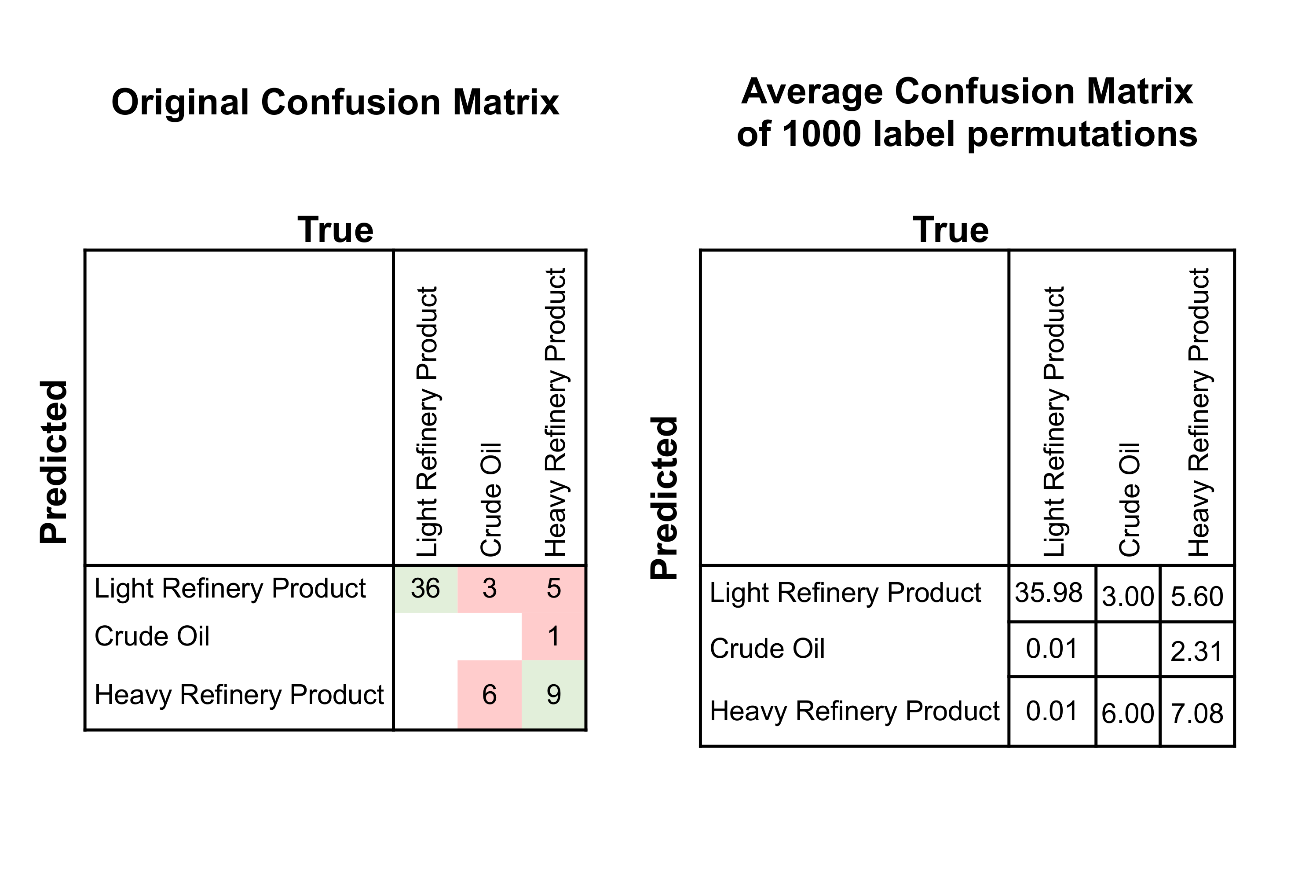

Supplement: S4 Fig — (DOCX) [file pone.0223517.s010.docx]
